# 5-Methylcytosine Related LncRNAs Reveal Immune Characteristics, Predict Prognosis and Oncology Treatment Outcome in Lower-Grade Gliomas

**DOI:** 10.3389/fimmu.2022.844778

**Published:** 2022-03-03

**Authors:** Jiheng Zhang, Nan Wang, Jiasheng Wu, Xin Gao, Hongtao Zhao, Zhihui Liu, Xiuwei Yan, Jiawei Dong, Fang Wang, Yixu Ba, Shuai Ma, Jiaqi Jin, Jianyang Du, Hang Ji, Shaoshan Hu

**Affiliations:** ^1^Department of Neurosurgery, The Second Affiliated Hospital of Harbin Medical University, Harbin, China; ^2^Department of Neurosurgery, Emergency Medicine Center, Zhejiang Provincial People’s Hospital, Affiliated to Hangzhou Medical College, Hangzhou, China; ^3^Department of Neurosurgery, Shandong Provincial Hospital Affiliated to Shandong First Medical University, Jinan, China

**Keywords:** 5-methylcytosine, long non-coding RNAs, immune, oncology treatment, lower-grade gliomas

## Abstract

5-Methylcytosine (m5C) methylation is an important RNA modification pattern that can participate in oncogenesis and progression of cancers by affecting RNA stability, expression of oncogenes, and the activity of cancer signaling pathways. Alterations in the expression pattern of long non-coding RNAs (lncRNAs) are potentially correlated with abnormalities in the m5C regulation features of cancers. Our aim was to reveal the mechanisms by which lncRNAs regulated the m5C process, to explore the impact of aberrant regulation of m5C on the biological properties of lower-grade gliomas (LGG), and to optimize current therapeutic. By searching 1017 LGG samples from the Cancer Genome Atlas and Chinese Glioma Genome Atlas, we first clarified the potential impact of m5C regulators on LGG prognosis in this study and used univariate Cox analysis and least absolute shrinkage and selection operator regression to explore clinically meaningful lncRNAs. Consequently, we identified four lncRNAs, including LINC00265, CIRBP-AS1, GDNF-AS1, and ZBTB20-AS4, and established a novel m5C-related lncRNAs signature (m5CrLS) that was effective in predicting prognosis. Notably, mutation rate, WHO class II, IDH mutation, 1p/19q co-deletion and MGMT promoter methylation were increased in the low m5CrLS score group. Patients with increased m5CrLS scores mostly showed activation of tumor malignancy-related pathways, increased immune infiltrating cells, and decreased anti-tumor immune function. Besides, the relatively high expression of immune checkpoints also revealed the immunosuppressed state of patients with high m5CrLS scores. In particular, m5CrLS stratification was sensitive to assess the efficacy of LGG to temozolomide and the responsiveness of immune checkpoint blockade. In conclusion, our results revealed the molecular basis of LGG, provided valuable clues for our understanding of m5C-related lncRNAs, and filled a gap between epigenetics and tumor microenvironment.

## Introduction

There are long-standing difficulties in the clinical management of lower-grade gliomas (LGG), which consist of diffuse low- and intermediate-grade gliomas (WHO grade II and III) (1). Due to invasiveness of the tumor cells and the consequent incomplete excision, patients frequently relapse and even malignantly transform to higher grades despite standard treatment (2). Although the emergence of molecular biomarkers such as IDH1 mutation and 1p/19q deletion are helpful for the diagnosis and treatment of LGG, there remains an urgent need for diagnosis, treatment, and assessment of prognosis of LGG (3, 4). Therefore, further characterizing the molecular underpinning of LGG will promote the current diagnosis and treatment of this lethal cancer.

Aberrant RNA modifications are associated with cancer cell survival, proliferation, invasion, and therapeutic resistance, and serve as potential therapeutic targets (5, 6). 5-methylcytosine (m5C) is a widespread RNA modification that functions to maintain RNA export, RNA stability and ribosome assembly by adding a methyl group to the carbon-5 position of a cytosine base. m5C is dynamically regulated by vital regulators including ‘writers’ (catalytic modification formation), ‘readers’ (recognition and binding of modified nucleotide) and ‘erasers’ (removal of modification), and participants in tumorigenesis (7–9). Recent findings suggest that the regulatory factor NSUN2, a member of the ‘writers’, promotes abnormally hypermethylation of m5C through the NSUN2/YBX1/m5C HDGF signaling pathway, which promotes the proliferation of uroepithelial cancer cells (10). Notably, deletion of NSUN5 results in a non-m5C methylation state at position C3782 of 28S rRNA, driving an overall suppression of protein synthesis, which contributes to long-term survival of glioblastoma patients (11). However, the role of m5C regulators, a hotspot for molecular research with great potential, in LGG remains obscure. Given the impact of aberrant expression and genetic alterations of m5C regulators on tumor malignant progression, a comprehensive analysis of them and their associated genes is warranted.

Long non-coding RNAs (lncRNAs) are essential in the modification of RNA. ZFAS1 regulates the activity of small nucleolar RNA-induced rRNA 2’-O-methylation through a ZFAS1-NOP58-SNORD12C/78-EIF4A3/LAMC2 signaling-dependent manner, thus promoting the proliferation and migration of colorectal cancer (CRC) cells (12). RNA-binding regulatory peptide, a 71 amino acid peptide encoded by LINC00266, can bind to IGF2BP [the ‘reader’ of N6-methyladenosine (m6A)] to enhance the m6A methylation of mRNA c-Myc, ultimately promoting CRC (13). In addition, the effects of lncRNAs on the tumor microenvironment (TME) have been widely reported, for example, high expression of LNC-EGFR in liver cancer binds to EGFR, stabilizes and maintains the RAS/ERK/AP1 signaling pathway, leading to Treg differentiation, cytotoxic T lymphocyte (CTL) suppression (14). And the mining of high-dimensional data has led to a new level of human understanding of tumor-immune-lncRNA interactions, such as the proposal of tumor immune subtypes and the revelation of their effects on immune cell infiltration (15, 16). Although lncRNAs have been shown to play a key role in LGG proliferation and differentiation, which can predict the prognosis of gliomas (17, 18). However, there is still a lack of understanding of m5C-related lncRNAs and how they interact with m5C in LGG. Therefore, an in-depth and comprehensive exploration of m5C-related lncRNAs may help to improve this situation, complement a gap between immuno-epigenetic-tumor, and provide new perspectives for cancer diagnosis and treatment.

In this perspective, we identified 13 m5C regulators with significantly altered expression in LGG which was of prognostic value. Accordingly, we identified four lncRNAs associated with m5C with prognostic value. These four lncRNAs were differentially expressed not only in different clinical subtypes of LGG, but also in multiple tumors by pan-cancer analysis. On this basis, we constructed the m5C-related lncRNA signature (m5CrLS) and stratified the m5CrLS scores to explore the different features of mRNA expression profiles, clinicopathological parameters, signaling pathways and gene mutations in LGG. After finding abnormalities in multiple immune-related pathways in the signaling pathways of the high and low m5CrLS score groups, we further explored and determined that increased LGG immune cells infiltration and reduced antitumor immune effect were associated with increased m5CrLS scores. Notably, the m5CrLS score also strongly predicted the efficacy of temozolomide (TMZ) and the therapeutic response to immune checkpoint blockade (ICB) in LGG patients. In conclusion, our analysis of m5C-related lncRNAs quantified the characteristics of LGG and provided a viable reference for optimizing the treatment of LGG.

## Materials and Methods

### Gene Expression Dataset

The mRNA expression profile of 105 normal brain tissues of GTEx was obtained from UCSC Xena (https://xena.ucsc.edu/). Transcriptomic expression profiles (FPKM normalized) and corresponding clinicopathological data (including IDH status, 1p/19q status, O(6)-methylguanine DNA methyltransferase (MGMT) promoter status, WHO classification, age, gender, and survival information) were retrieved from The Cancer Genome Atlas (TCGA) database (https://portal.gdc.cancer.gov/). Samples without survival information were excluded, and 504 LGG samples were retained for further analysis. In addition, the expression profile of 513 LGG samples as well as corresponding demographics (**Table 1**) were downloaded from the Chinese Glioma Genome Atlas (CGGA) database (http://www.cgga.org.cn/). Datasets were subjected to the R package ‘limma’ for removing batch effect. The mRNA-seq datasets (FPKM normalized) of 33 types of cancers were also retrieved from the TCGA GDC project in UCSC Xena data portal. LGG somatic mutation data in UCSC Xena were analyzed and visualized using the R package ‘maftools’ (19).

**Table 1:** The clinical characteristics of LGG patients in the TCGA and CGGA datasets.

Characteristic	TCGA	CGGA	No. of Patients
**Total sample**	504	513	1017
**Age (years)**			
< =50	309	434	743
>50	140	79	219
Unknown	55		55
**Gender**			
male	251	296	547
female	198	217	415
Unknown	55		55
**Grade**			
WHO II	213	238	451
WHO III	236	275	511
Unknown	55		55
**IDH status**			
Mutant	410	389	799
Wildtype	94	124	218
**1p/19q codeletion**			
codel	166	162	328
no-codel	338	351	689
**MGMT promoter status**			
Methylated	416	246	662
Unmethylated	88	172	260
Unknown		95	95
**Histology**			
astrocytoma	165		165
oligodendroglioma	172		172
oligoastrocytoma	112		112
Unknown	55		55

### m5C Regulatory Genes and Protein-Protein Interaction (PPI) Networks

13 confirmed m5C regulatory factors were selected from the available literature, including DNMT1, DNMT3A, DNMT3B, ALYREF, NOP2, NSUN2, NSUN3, NSUN4, NSUN5, NSUN6, NSUN7, TET2, TRDMT1 (20, 21). The R package ‘limma’ was used to integrate and remove batch effects from TCGA and GTEx data, as well as to analyse differences in 13 regulators. The p value < 0.05 was considered significant.

The Search Tool for Interacting Genes (STRING) database (https://string-db.org/) was used to construct PPI interaction networks based on methods including scientific text mining, extraction of experimental data, and calculation of genomic features (22). The potential interactions between 13 m5C regulators were explored by setting the minimum required interaction scores on the STRING online website to a high confidence level (> 0.7). The cytoscape software (version 3.7.2) was used to visualize the PPI network. Furthermore, hub genes were identified using maximal clique centrality (MCC) computing method with the cytohubba plugin (23).

### m5C-Related LncRNAs

The Genome Reference Consortium Human Build 38 (GRCh38) annotation file was obtained from the GENCODE website (https://www.gencodegenes.org/human/) to annotate the TCGA and CGGA datasets, and 14,142 and 1,004 lncRNAs were annotated, respectively. Pearson correlation analysis was used to screen out 222 m5C-related lncRNAs from TCGA and 81 m5C-related lncRNAs from CGGA, based on 13 m5C regulatory factors. Absolute value of correlation coefficient > 0.5 and p value < 0.01 were set as cutoff.

### Determine m5CrLS

Firstly, all lncRNAs associated with m5C were screened by univariate Cox regression analysis, and 107 and 46 lncRNAs with prognostic value were obtained in the TCGA and CGGA datasets, respectively. The least absolute shrinkage and selection operator (LASSO) regression analysis was performed on six lncRNAs co-expressed in both datasets using the package ‘glmnet’ to screen out biomarkers for further development m5CrLS. The following equation was used to calculate the m5CrLS score for LGG:


$$m5CrLS\;score=\sum_{i=0}^{n}\left(Regression\;Coeffecient*Expression\right)$$

The samples were split into high and low m5CrLS score groups by the median value.

### Functional Enrichment Analysis

Differentially expressed genes (DEGs) between low and high m5CrLS score groups were calculated using the R package ‘limma’ based on the TCGA dataset with the criteria of | log2(FC) | > 1, p < 0.05, and FDR < 0.05. DEGs up-regulated [log2(FC) > 0] in the high m5CrLS score group were selected as candidates for Gene Ontology (GO) analysis, including cellular component (CC), molecular function (MF), biological process (BP), and Kyoto Encyclopedia of Genes and Genomes (KEGG) based on the R package ‘clusterProfiler’ and ‘enrichplot’ for visualization.

The relative activation of classical tumor-associated pathways in high and low m5CrLS score groups was characterized using single-sample gene set enrichment analysis (ssGSEA). The signature genes used for the calculations were derived from a summary of recent researches (24, 25). In the analysis, ssGSEA scores were normalized to a unit distribution, where 0 was the minimum value of activation for each pathway and 1 represented the maximum.

### Immunoinformatic Analysis

‘ESTIMATE’ algorithm was used to assess the content of immune and stromal cells in the TME of each sample, which was expressed in the form of four scores: immune score, matrix score, estimated score and tumor purity (26).

The fraction of B-cell, CD4^+^ T-cell, CD8^+^ T-cell, dendritic cell, macrophage, and neutrophil infiltration was estimated using the TIMER2.0 webtool (http://timer.cistrome.org/) based on mRNA expression profiles (27). The R package ‘limma’ was used for differential analysis and Spearman correlation analysis was performed to calculate the correlation between the m5CrLS score and the fraction of 6 immune infiltrations.

The ssGSEA algorithm was performed to further quantify the infiltration of 28 immune cells in the TME. The characteristic genes for each type of immune cell were summarized by JIA et al. (28). The ssGSEA scores distributed from 0 and 1 represented the minimum and maximum of each immune cell infiltration abundance, respectively.

Tumor Immunophenotype Profiling (TIP) (http://biocc.hrbmu.edu.cn/TIP/) is a web-based tool that allows convenient and rapid analysis and visualization of the extent of tumor-infiltrating immune cells and the 7 phase events of anti-cancer immunity, including tumor cell antigen release (step 1), cancer antigen presentation (step 2), stimulation and activation (step 3), immune cell transfer to the tumor (step 4), immune cell infiltration (step 5), T cell recognition of cancer cells and killing of cancer cells (steps 6, 7) (29). In view of this, the antitumor immune function activity of LGG was quantified by TIP and further analyzed for differences between high and low m5CrLS score groups.

### Prediction of ICB Responsiveness

The Tumor Immune Dysfunction and Exclusion (TIDE) (http://tide.dfci.harvard.edu) web platform integrates published immune checkpoint blockade (ICB) trials to predict clinical response to ICB (30). Both datasets were assessed by the TIDE algorithm and samples with TIDE scores below a threshold (default value of 0) were set to be responsive to ICB treatment.

### Statistical Analysis

All statistical analysis was performed on R (version 4.1.1). Kaplan-Meier (K-M) analyses and log-rank statistical tests were used to compare overall survival (OS) between groups. Survival analysis grouping based on 13 m5C regulators and four lncRNAs was divided according to the median of each gene expression. In addition, the median value of m5CrLS scores from TCGA dataset was used as node for survival analysis between m5CrLS groups as well as determination of sensitivity to TMZ and radiotherapy treatment. Receiver operating characteristic (ROC) curves and the corresponding area under the curve (AUC) values were used to evaluate the predictive power of m5CrLS scores established based on the expression of four biomarkers for prognosis. Univariate and multifactorial Cox regression analyses were used to assess the independent prognostic value of the m5CrLS and 13 m5C regulators. Wilcoxon test was performed to compare intra-subgroup differences in clinicopathological subtype, immune cell infiltration, ssGSEA score, the expression of immune checkpoint, and the TIP score. Kruskal test was used to compare differences between histopathological subtypes (astrocytoma, oligodendroglioma, oligoastrocytoma). A chi-square test was used to compare gender, age, WHO grade, IDH status, 1p/19q status, O(6)-methylguanine DNA methyltransferase (MGMT) promoter methylation status, and distribution characteristics of histopathological subtypes between low and high m5CrLS score groups. The difference in response to ICB treatment between low and high m5CrLS score groups was assessed using the fisher test.

## Results

### Aberrant m5C Methylation

**Figure 1:**
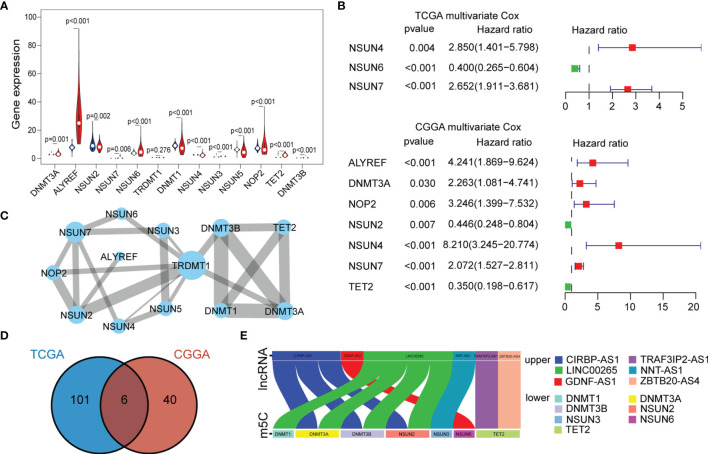
**(A)** The expression of individual m5C methylation regulators (blue represents the normal brain tissue and red represents LGG). **(B)** Multivariate Cox regression analysis of 13 m5C regulators (genes with p < 0.05 were exhibited). **(C)** PPI networks show the interaction between different m5C regulators (13 nodes, 23 edges). The width of the linkage was proportional to the connectivity degree, and node size was positively correlated with its centrality. **(D)** Venn diagram exhibiting the 6 lncRNAs expressed in the TCGA and CGGA datasets selected by univariate Cox analysis. **(E)** Correlations between the six lncRNAs and corresponding m5C regulators based on the TCGA dataset.

To investigate the expression of 13 m5C regulatory factors in LGG, normal and tumor samples from the GTEx and TCGA databases were integrated. As a result, DNMT3A, DNMT3B, ALYREF, NSUN3, NSUN6, NSUN7, and TET2 were significantly up-regulated in tumor samples (p < 0.05), and NOP2, NSUN2, NSUN4, NSUN5, and DNMT1 regulators were significantly down-regulated (p < 0.05), while TRDMT1 was the only regulator without significant change in expression (p = 0.276) (**Figure 1A**). Then, K-M analysis was performed to explore the expression of these m5C regulators on LGG survival. The results showed that patients with decreased expression of DNMT3A, DNMT3B, DNMT1, NOP2, NSUN4, and NSUN7 had improved OS, which was also validated by the CGGA dataset (**Figures S1A, B****)**. The univariate and multivariate Cox regression analysis showed that NSUN4 and NSUN7 were independent risk factors for LGG based on both datasets (**Figures 1B**, **S1C**).

To further characterize the interactions between these m5C regulators, a protein-protein interaction (PPI) network was constructed (**Figure 1C**). As a result, TRDMT1 scored highest and was the hub gene in the network, and genes with their corresponding centrality weights were collated as **Table S1**. Subsequently, correlation analysis found that TET2 had the highest negative correlation with NSUN5 (cor = -0.44, p < 0.05) and the highest positive correlation with NSUN3 (cor = 0.68, p < 0.05) (**Figure S1D**). Overall, these results demonstrated the LGG-related m5C regulators and their interactions.

### Identification of m5C-Related LncRNAs With Prognostic Significance

To further explore the impact of lncRNAs on the m5C regulatory patterns of LGG, we firstly conducted the Pearson correlation analysis between the expression of lncRNAs and m5C regulators. With | cor | > 0.5 and p < 0.01 as the threshold, a total of 222 and 81 m5C-related lncRNAs were identified in TCGA and CGGA samples. With univariate Cox analysis, 107 and 46 m5C-related lncRNAs retained prognostic significance in TCGA and CGGA samples (**Tables S2**, **S3**). We noted that LINC00265, CIRBP-AS1, GDNF-AS1, ZBTB20-AS4, NNT-AS1 and TRAF3IP2-AS1 were all identified as m5C-related lncRNAs in both datasets (**Figure 1D**). Also, the correlations between the expression of these six lncRNAs and m5C regulators were strong (cor > 0.5) (**Figures 1E**, **S1E** and **Tables 2**, **S4**).

**Table 2:** The correlations between m5C regulators and lncRNAs based on the TCGA dataset.

LncRNA	m5C	Correlation coefficient	p-value	Direction
CIRBP-AS1	DNMT3A	0.5288	1.92E-39	positive
CIRBP-AS1	DNMT3B	0.5493	5.05E-43	positive
CIRBP-AS1	NSUN2	0.5795	8.94E-49	positive
GDNF-AS1	NSUN6	0.5410	1.49E-41	positive
LINC00265	DNMT1	0.5259	5.84E-39	positive
LINC00265	DNMT3A	0.5666	2.97E-46	positive
LINC00265	DNMT3B	0.6056	2.93E-54	positive
LINC00265	NSUN2	0.5323	4.91E-40	positive
NNT-AS1	NSUN3	0.5115	1.31E-36	positive
TRAF3IP2-AS1	TET2	0.5098	2.43E-36	positive
ZBTB20-AS4	TET2	0.5361	1.06E-40	positive

**Figure 2:**
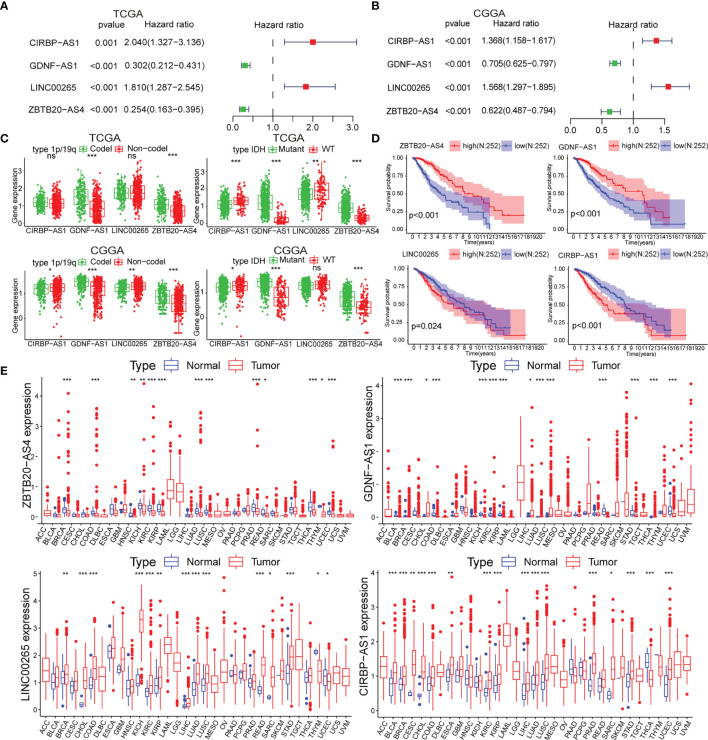
**(A, B)** Univariate Cox analysis for the four m5C-related lncRNAs. **(C)** Differential expression of the four m5C-related lncRNAs in clinical subgroups (including 1p/19q co-deletion or no-co-deletion, and IDH mutant or wildtype). **(D)** K-M curves of the four m5C-related lncRNAs based on the TCGA dataset. **(E)** Pan-cancer analysis of the four m5C-related lncRNAs. (ns, non-significant, *p < 0.05, **p < 0.01, and ***p < 0.001).

To identify biomarkers, we identified four of the six lncRNAs with significant prognostic significance by LASSO regression analysis, namely LINC00265, CIRBP-AS1, GDNF-AS1 and ZBTB20-AS4 (**Figure S2A**). Particularly, GDNF-AS1 and ZBTB20-AS4 were protective factors, while LINC00265 and CIRBP-AS1 were risk factors (**Figures 2A, B****)**. Taking into account the clinicopathological features of LGG, the expression of GDNF-AS1 and ZBTB20-AS4 was significantly increased in samples of 1p/19q co-deletion and IDH mutation in both datasets (p < 0.001), and the expression of CIRBP-AS1was decreased in samples with the IDH mutation (**Figures 2C**, **S2B**). K-M curves showed that LGG with increased expression of GDNF-AS1 and ZBTB20-AS4 had improved survival, whereas patients with elevated expression of LINC00265 and CIRBP-AS1 had poor OS, in line with the result of Cox regression analysis (**Figures 2D**, **S2C**). In addition, we analyzed the expression of the four m5C-related lncRNAs in 33 tumors using a pan-cancer analysis. Compared to normal tissues, ZBTB20-AS4, CIRBP-AS1, GDNF-AS1, and LINC00265 were differentially expressed in a variety of tumors, including BRCA, COAD, KIRC, KIRP, LUAD, LUSC, and UCEC, suggesting that these m5C-related lncRNAs may be conserved in the tumor progression (**Figure 2E**). In summary, these results suggested that these four m5C-related lncRNAs were potential candidates for the development of a reliable prognostic model.

### Construction and Validation of the m5CrLS-Based Stratification

**Figure 3:**
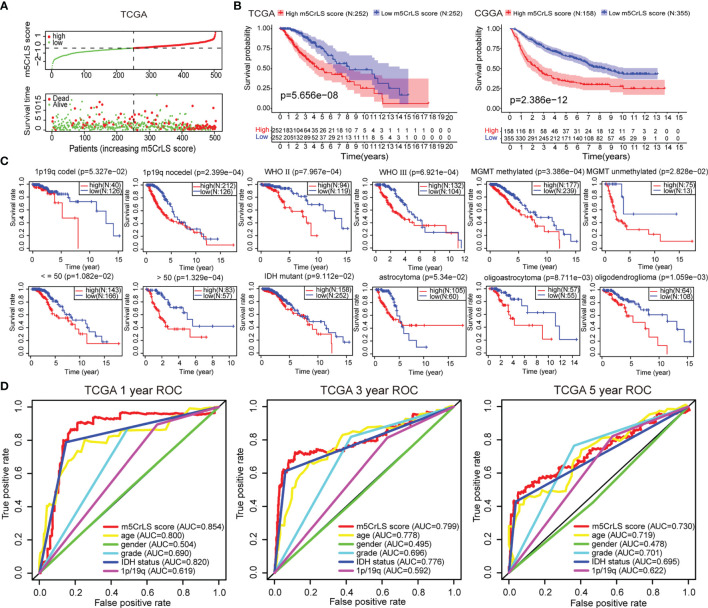
**(A)** The distribution plots of the m5CrLS score and survival in the TCGA. **(B)** K-M curves showing that the survival difference between the high and low m5CrLS score groups (p < 0.001). **(C)** K-M curves of the m5CrLS-based stratification in multiple TCGA clinical subgroups. **(D)** ROC curves exhibiting the time-dependent predictive value of the m5CrLS score.

**Figure 4:**
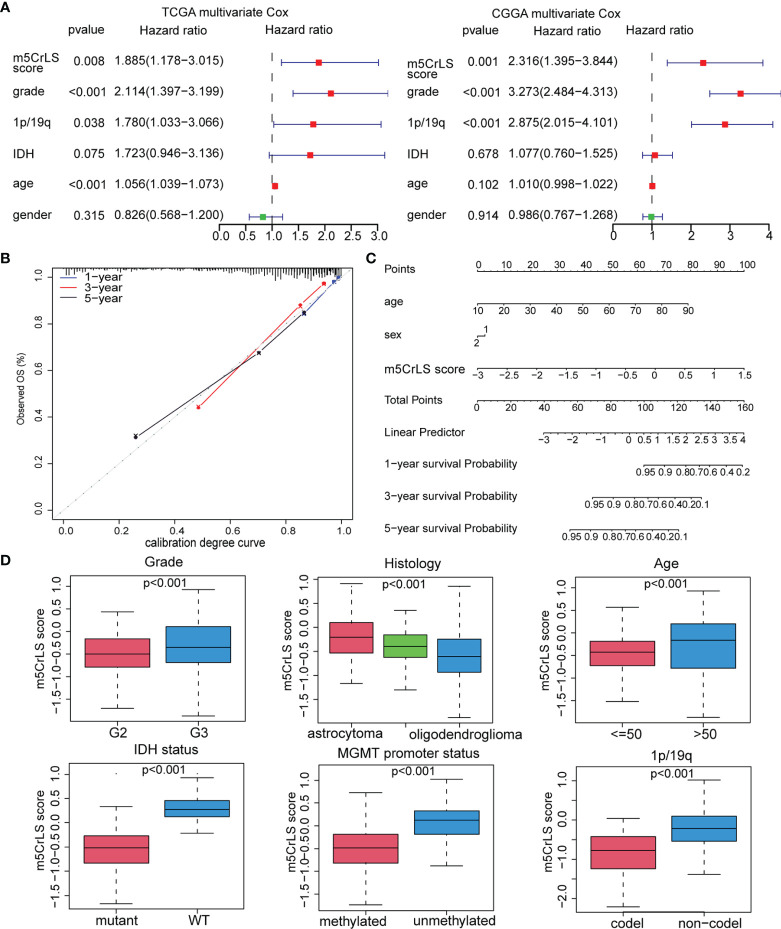
**(A)** Multivariate Cox regression analysis of the TCGA and CGGA dataset. **(B)** Calibration chart for predicting the probability of survival at 1-, 3-, and 5-year in the TCGA dataset. **(C)** Construction of nomogram graph based on m5CrLS score, age, and gender. **(D)** The relationship between the m5CrLS scores and clinicopathological subgroups of TCGA dataset.

Next, we constructed the m5CrLS using the four m5c-related lncRNAs and their LASSO regression coefficients to quantify individual differences among LGG samples (**Figure S3A**). The distribution of m5CrLS score with survival time and status showed that the mortality increased with the score (**Figures 3A**, **S3B**). K-M curves suggested that the low m5CrLS score group had a significantly improved OS (p < 0.001) (**Figure 3B**). Notably, the m5CrLS group predicted prognosis was independent of age, WHO tumor grade, MGMT promoter status, and IDH status (**Figures 3C**, **S3C, D**). The ROC curves showed a robust time-dependent predictive power of the m5CrLS, with the 1-year AUC values of 0.854 and 0.740, the 3-year AUCs of 0.799 and 0.724, and the 5-year AUCs of 0.730 and 0.685, in the TCGA and CGGA datasets, respectively which was an improved predictor than clinical characteristics including age, gender, grade, IDH status and 1p/19q status (**Figures 3D**, **S3E**). The results of univariate [TCGA hazard ratio (HR) = 3.864, 95% confidence interval (CI) =2.825 - 5.286, p < 0.001; CGGA HR = 4.151, 95% CI = 2.881 - 5.980, p < 0.001] and multivariate Cox analysis (TCGA HR = 1.885, 95% CI = 1.178 – 3.015, p = 0.008; CGGA HR = 2.316, 95% CI = 1.395 - 3.844, p = 0.001) showed that the m5CrLS score was an independent risk factor (**Figures 4A**, **S4A**). The accuracy of the prognostic model can be determined by the calibration degree curve (**Figure 4B**). Moreover, to render the m5CrLS score a clinically applicable quantitative standard, a nomogram was constructed (**Figure 4C**).

In addition, the differential expression of the four prognostic m5C-related lncRNAs and their associated clinical features were exhibited. And samples of WHO grade II, 1p/19q co-deletion, IDH mutation, and MGMT promoter methylation were enriched in the low m5CrLS score group (p < 0.05) (**Figures S4B, C**). Particularly, WHO grade II, 1p/19q co-deletion, IDH mutation, MGMT promoter methylation, and oligodendroglioma had decreased m5CrLS scores (**Figures 4D**, **S5A**). The results of principal component analysis (PCA) in high and low m5CrLS score groups showed that those had different distributions based on the expression of prognostic biomarkers, all m5C-related lncRNAs, 13 m5C regulators, and all genes, suggesting significant differences in molecular and m5C methylation characteristics between patients in the two groups (**Figure S5B**).

### Functional Enrichment Analysis

**Figure 5:**
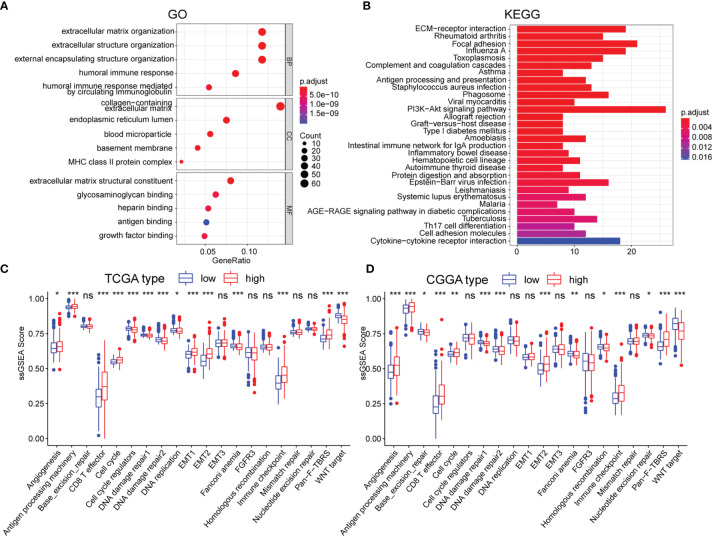
**(A, B)** Functional annotation based on 520 up-regulated in the high m5CrLS score group, using GO terms of BP, CC, MF, and KEGG pathway. The abnormality of tumor-related pathways is based on **(C)** TCGA dataset and **(D)** CGGA dataset. (* p < 0.05, ** p < 0.01, and *** p < 0.001; ns, non-significant).

To further uncover the molecular underpinning of m5CrLS stratification, functional enrichment analysis was performed. Firstly, 649 differentially expressed genes were identified and 520 genes were significantly up-regulated in high m5CrLS score LGG (**Table S5**). GO and KEGG enrichment analysis revealed that 520 up-regulated genes were mainly enriched in the extracellular matrix organization, extracellular structural organization, ECM-receptor interaction, and PI3K-AKT signaling pathway. Notably, high and low m5CrLS score patients have multiple immune response processes in different states of activation, such as humoral immune response, MHC class II protein complex (**Figures 5A, B****)**. In addition, we selected 20 classical tumor-associated pathways whose activation tended to correlate with the degree of tumor malignancy (24, 25). As a result, the high m5CrLS score group showed an increased ssGSEA score of angiogenesis, cell cycle, epithelial-mesenchymal transition (EMT), pan-fibroblast transforming growth factor-beta (Pan-F TBRS). In contrast, the low m5CrLS score group had increased ssGSEA score in the DNA damage repair, WNT target pathway (**Figures 5C, D****)**. The above results may suggest that tumor cells in the high m5CrLS score group were more malignant and aggressive.

### Differences in the Immune Infiltration

**Figure 6:**
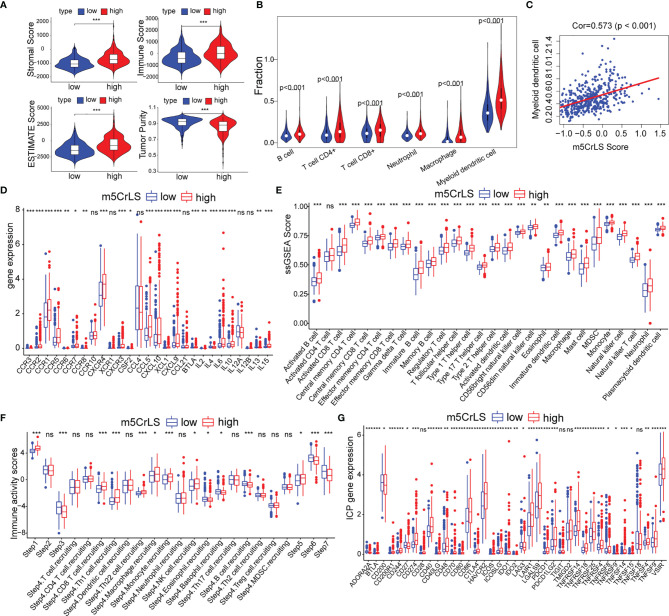
**(A)** Stromal score, immune score, estimate score, tumor purity of TCGA dataset. **(B)** Relationship between m5CrLS-based stratification and TIMER2.0 of 6 immune cells. **(C)** Correlation of myeloid dendritic cells with m5CrLS scores. **(D)** Chemokines and cytokines associated with dendritic cells were differentially expressed between high and low m5CrLS score in TCGA dataset. **(E)** Differences in 29 immune cells between high and low m5CrLS score patients of TCGA dataset. **(F)** 7 steps of the anti-tumor immune response analyzed by TIP. **(G)** The expression level of immune checkpoint in TCGA dataset. (ns, non-significant. *p < 0.05, **p < 0.01, and ***p < 0.001).

Given that the differences in immune-related biological processes and signaling pathways between high and low m5CrLS score groups, the immune microenvironment was further explored. The stromal score, immune score, estimate score, and tumor purity were calculated based on the R package ‘ESTIMATE’. As a result, the high m5CrLS score group consistently scored higher, indicating a possible increase in the immune and stromal infiltration (**Figures 6A**, **S6A**). Thus, the fraction of six immune cells, including B cells, CD4^+^ T cells, CD8^+^ T cells, neutrophils, macrophages, and myeloid dendritic cells were inferred by TIMER2.0. Consistently, the infiltration of these immune cells was significantly increased in the high m5CrLS score group (**Figure 6B**). Spearman correlation analysis suggested that myeloid dendritic cells had the highest positive correlation with m5CrLS score (cor = 0.573, p < 0.001). Furthermore, myeloid dendritic cells showed a strong positive correlation with immune infiltration of neutrophils (cor = 0.86) (**Figures 6C**, **S6B, C**). Chemokines CCR1, CCR2, CCR3, CCR6, CCR5, CCL5, CXCL10, and XCL1 showed high expression in the high score group of m5CrLS to promote the migration of dendritic cells. In addition, the high expression of CCR7, CCL21 inhibited the function of dendritic cells and hindered the killing effect of T cells on tumor cells (31–33). The cytokine IL10, which showed high expression in the high m5CrLS score, could inhibit the release of IL12A from dendritic cells, thus causing anti-tumor immunosuppressive effects (34). In addition, IL6 and IL15 showed high expression in the m5CrLS high subgroup (**Figures 6D**, **S6D**). For validation, immune infiltration was also estimated using the ssGSEA algorithm and similar results were yielded that 24 out of 28 immune cells scored higher in high m5CrLS score patients (**Figures 6E**, **S6E**). To further explore the ability of anti-tumor immune response in high and low m5CrLS score groups, we quantified the activity of 7 steps of LGG anti-tumor immune response based on TIP. The results showed that the low m5CrLS score group scored higher in step 3, step 6, and step 7, while the high m5CrLS score group scored higher in the release of step 1, and step 5 **(****Figure 6F****)**. The above results suggest that the stratification analysis based on m5CrLS can identify the antitumor immune activity of LGG to some extent.

Given the impact of immune checkpoints (ICPs) on anti-tumor immunity, we investigated the expression of those ICPs. Tumor necrosis factor superfamily ligands (TNFSF) and receptors (TNFRSF), including TNFSF9, TNFSF4, TNFSF15, TNFSF14, CD40, CD70, CD40LG, TNFRSF4, TNFRSF8, TNFRSF9, TNFRSF14, TNFRSF18, and TNFRSF25 were up-regulated in the high m5CrLS score group in both datasets. Programmed cell death protein-1 (PD-1) and two binding ligands, PD-L1 and PD-L2, were highly expressed in the high m5CrLS score group, indicating T-cell depletion and decreased effector function (35). CTLA4 was highly expressed in the high m5CrLS score group and could bind to equally highly expressed CD86 and CD80 to transduce T-cell suppressor signals and promote the suppressive function of Treg cells (36). TIM-3 (HAVCR2) and LGALS9 were significantly associated with T-cell depletion and impaired function, and low expression in the low m5CrLS score group suggested a stronger autoimmune and antitumor immune response than in the high m5CrLS score group (37). In addition, high expression of ICOS, ICOSLG, and IDO1 in high m5CrLS score patients contributed to Treg cell aggregation (38, 39). high expression of LAIR1 suppressed CD8^+^ T cell activity in high m5CrLS score patients (40). High expression of CD48, CD244 (41), CD200R1 (42), and BTLA (43) also had a suppressive effect on anti-tumor immunity **(****Figures 6G**, **S6F****)**.

### Relationship between m5CrLS Stratification and Mutational Status

**Figure 7:**
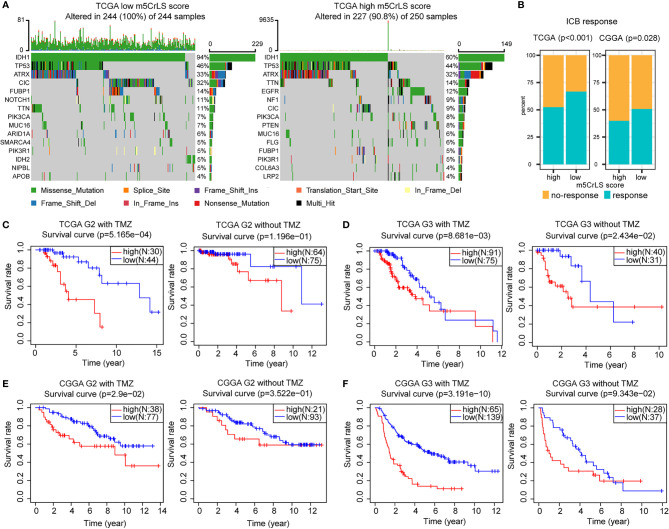
**(A)** Differences in mutations between high and low m5CrLS score groups (the top 15 mutated genes). **(B)** Predicting the relationship between m5CrLS-based stratification and ICB responsiveness. K-M curves of **(C)** grade II and **(D)** grade III patient receiving TMZ or without TMZ based on TCGA dataset. K-M curves of **(E)** grade II and **(F)** grade III patient receiving TMZ or without TMZ based on CGGA dataset.

The tumor immune microenvironment was also associated with somatic mutation rates (44). The analysis of LGG mutation information allowed us to better understand patients with different m5CrLS. The mutation rate in the low m5CrLS score group was as high as 100%, while the mutation rate in the high m5CrLS score group was 90.8%. This implies a high release of tumor neoantigens and more effective anti-cancer immune activity in the low m5CrLS score group with higher somatic mutation rates (45–47). In addition, the two groups differed in terms of high IDH1 mutations (94% in the low m5CrLS score group and 60% in the high m5CrLS score group). It has been noted that IDH1 mutations suppress the infiltration of immune cells such as macrophages, microglia, and neutrophils (48). This further explains the lower number of cancer-promoting immune cells in patients with low m5CrLS scores. Interestingly, PTEN was mutated at a frequency of 8% in the high m5CrLS score group but did not appear in the top 15 mutated genes of the low m5CrLS score group in terms of mutation frequency. Mutations in PTEN result in its loss of tumor suppressive function (49). Notably, increased PTEN mutations and excessive activation of PI3K-AKT were associated with ICB treatment resistance mechanisms (50) (**Figure 7A**).

### Prediction of the Efficacy of Therapy

ICB has achieved surprising results in the treatment of tumors. Previously available studies have highlighted the impact of RNA modification on ICB treatment. With this in mind, we employed the TIDE algorithm based on the stratification of m5C-related lncRNAs to assess the efficacy of LGG on ICB treatment. The results showed that there was a significant difference in the response to ICB treatment between the two groups (p < 0.05), and that low m5CrLS score patients were more sensitive to ICB treatment (**Figure 7B**).

Next, the association of TMZ efficacy with m5CrLS score groups was examined. K-M analysis showed that in LGG of grade II who received TMZ, OS was significantly higher in the low m5CrLS score group. In contrast, there was no statistical difference between the high and low m5CrLS score groups that did not receive TMZ (**Figures 7C, E****)**. Similar results were also yielded in the samples of grade III that the low m5CrLS score group predicted improved OS in patients who received TMZ (**Figures 7D, F****)**.

In addition, the association between the stratification analysis and the efficacy of radiotherapy was evaluated. In the TCGA dataset, low m5CrLS score patients of grade II had a significant survival benefit after receiving radiotherapy, and such result can almost be validated by the CGGA cohort **(****Figures S7A, C**). There was no significant difference in OS for patients in the high and low m5CrLS score groups of grade II who did not receive radiotherapy. In grade III, both datasets showed improved OS for the low m5CrLS score group regardless of whether they received radiotherapy or not (**Figures S7B, D**).

## Discussion

m5C methylation is regulated by “writer, reader, and eraser” regulators that affect tumor progression under abnormal conditions (51, 52). A comprehensive analysis of m5C regulators and their associated lncRNAs can help clarify the role of m5C modification patterns on LGG tumor cells and guide more effective individualized therapeutic strategies. In this study, we first revealed the aberrant expression levels of 13 m5C regulators in LGG and their prognostic implications. After identifying four m5C-related lncRNAs with prognostic value, we constructed a novel m5CrLS to systematically assess individual differences in LGG. Stratified analysis of the m5CrLS suggested aberrant expression patterns and m5C methylation status in both groups. In addition, the high m5CrLS score was characterized by activation of tumor malignancy-related pathways and poor prognosis. m5CrLS also distinguished different immune infiltration and anti-tumor immunity in LGG patients, where the high m5CrLS score subgroup had higher levels of immune cell infiltration and poorer anti-tumor immunity. Notably, the low m5CrLS score group was not only more sensitive to TMZ treatment, but also had a higher response to ICB treatment.

Increasing evidence suggests that aberrant expression of lncRNAs may be associated with the development of multiple cancers and could be a potential therapeutic target (53, 54). lncRNA H19 modified by m5C methylation promotes hepatocarcinogenesis and progression through recruitment of oncoprotein G3BP1 (55). Recently, deep bioinformatics mining of high-throughput data revealed that m5c-related lncRNAs have potential effects on immune infiltration and can be used as multiple tumor prognostic markers and therapeutic targets (56, 57). To our knowledge, this is the first exploration of m5C-related lncRNAs in LGG, yielding the prognostic biomarkers LINC00265, CIRBP-AS1, GDNF-AS1 and ZBTB20-AS4. LINC00265 acts as an endogenous sponge that enhances the expression of ZMIZ2, which in turn recruits the enzyme USP7 to deubiquitinating and stabilize oncogenic β-catenin, thereby promoting colorectal tumorigenesis (58). In addition, LINC00265 can act as an m6A-associated lncRNA that affects the prognosis of LGG (59). Triple-negative breast cancer patients with low expression of CIRBP-AS1 have a better prognosis (60). GDNF-AS1 is also associated with ferroptosis and has an impact on the prognosis of glioma (61). However, ZBTB20-AS4 has been rarely reported. We hope that our findings will help identify lncRNAs associated with m5C methylation and thus provide insight into their potential role in LGG tumorigenesis and progression.

Some studies identified a potential relationship between m5C modulators and immune infiltration (20, 62). Furthermore, lncRNAs perform a critical function in the TME, such as promoting M2-type macrophage polarization, downregulating tumor-associated antigens, and inhibiting CTL function (63–65). Notably, few have combined the two to explore the complex immune microenvironment of LGG. We linked m5CrLS to the distribution of infiltrating immune cells in LGG for the first time. Surprisingly, in our study, high m5CrLS score patients exhibited a large infiltration of anti-tumor immune cells in addition to many immune cells with suppressive effects on tumor cytotoxicity, such as Tregs, MDSCs, and tumor-associated macrophages. However, these did not prolong the OS of patients. This was in line with many studies (62, 66). Differently, we went further to quantify the antitumor immune process and found that the activation status of the three steps of T-cell initiation and activation, T-cell recognition of cancer cells and killing of cancer cells was lower in the high m5CrLS score group than in the low m5CrLS score group. Combined with the analysis of TNFSF, chemokines, and cytokines, this also explains to some extent the lower number of immune infiltrating cells and stronger anti-tumor immune function in patients with low m5CrLS scores. Our study demonstrated the importance of m5C modification-related lncRNAs in shaping a different immune microenvironment landscape. Thus, a comprehensive assessment of m5C modification patterns distinguishes different features of tumor-infiltrating immune cells and antitumor immune processes in LGG, complementing the immune RNA modification-lncRNA relationship in LGG.

The effects of ICPs on T-lymphocyte activity and effector function were well defined (67). In addition, PD-1 and PD-L1 expression was also affected by RNA modification. Knockdown of FTO increases m6A methylation of PD-1 in melanoma cells, while deletion of ALKBH5 (the ‘eraser’ of m6A) enriches the 3’UTR region of PD-L1 mRNA with m6A modifications, thereby promoting the degradation of PD-1 and PD-L1 in a YTHDF2-dependent manner (68, 69). Aberrant activation of ICPs in the m5CrLS high score population interferes with antitumor immune function, leading to immune evasion of tumor cells (70). Notably, our analysis of ICPs not only re-illustrated the immune status of LGG after stratification, but also suggested a potential link between ICPs and m5CrLS, complementing the molecular mechanisms affecting the expression of multiple immune checkpoints in LGG. However, studies on the effects of m5C modifications on ICPs have not been seen. Although our study distinguished the expression of ICPs in patients with different m5CrLS, it also did not reveal the specific mechanisms that induce ICPs. However, we hope that our study can provide an idea for the study of RNA modifications and ICPs.

In addition, the m5CrLS-based stratification has the potential to screen ICB responders and predict radiotherapy efficacy. Anti-PD-1 ICB therapy was enhanced by inhibition of ALKBH5 *via* the m6A pathway, which may also be related to the inhibition of Treg accumulation (71). In addition, high-throughput data analysis also showed that m6A was closely associated with ICB treatment, where EMT played an important role in treatment resistance (72). In this work, we found that m5CrLS reveals increased Treg accumulation, angiogenesis, and EMT activation related. These abnormal states may mediate therapeutic resistance to ICB and affect LGG individual precision immunotherapy. TMZ is used as adjuvant therapy after glioma surgery and has significantly improved patient survival, but most patients unfortunately develop treatment resistance (73, 74). Deletion of the m5C methyltransferase NSUN2 results in skin tumor cells being killed more effectively by chemotherapeutic agents such as 5-fluorouracil or cisplatin (75). However, the impact of m5C methylation or m5C modulators on TMZ treatment is unclear. Our m5CrLS shows a strong sensitivity to the outcome of TMZ treatment, probably since most low m5CrLS score patients possess MGMT promoter methylation, which blocks the synthesis of O6-methylguanine and thus increases the sensitivity of tumors to the cytotoxic effects induced by TMZ (74). In addition, aberrant activation of the EMT pathway in the high m5CrLS score group also contributes to resistance to TMZ treatment (76).

In summary, we constructed a novel m5CrLS that can comprehensively evaluate the different expression patterns of individual patients and provided new insights into the LGG immune microenvironment and a solid foundation for it. However, it is worth noting that our study also has some limitations. We should further confirm the m5C modification sites or specific mechanisms on the four lncRNAs using cellular and tissue experiments, and further validate them against tumor immune function and immune infiltrating cells. In addition, our development of m5CrLS needs to be further validated in prospective studies and multicenter clinical trials. We will incorporate these efforts into future studies.

## Data Availability Statement

The datasets presented in this study can be found in online repositories. The names of the repository/repositories and accession number(s) can be found in the article/**Supplementary Material**.

## Author Contributions

JZ, NW, HJ, and SH conceived and designed the study. XG and JW collected the data. JZ, HZ, ZL, and XY provided analytical technical support. NW, JWD, FW, YB, SM, and JJ participated in the production of charts and pictures. JZ and NW drafted the manuscript. JZ, NW, JW, HJ, JYD, and SH revised the manuscript. All authors contributed to the article and approved the submitted version.

## Funding

This work was funded by the National Natural Science Foundation of China (No. 61575058).

## Conflict of Interest

The authors declare that the research was conducted in the absence of any commercial or financial relationships that could be construed as a potential conflict of interest.

## Publisher’s Note

All claims expressed in this article are solely those of the authors and do not necessarily represent those of their affiliated organizations, or those of the publisher, the editors and the reviewers. Any product that may be evaluated in this article, or claim that may be made by its manufacturer, is not guaranteed or endorsed by the publisher.

## Supplementary Material

Supplementary Figure 1 K-M curves showing the survival differences between m5C regulators in the high- and low-expressed groups. The median expression was used as the cut-off. **(A)** the TCGA dataset, and **(B)** the CGGA dataset. **(C)** Univariate Cox regression results for 13 m5C regulators (p < 0.05 was exhibited). **(D)** Correlation between the 13 m5C methylation regulators based on TCGA dataset. **(E)** Correlation between LINC00265, CIRBP-AS1, GDNF-AS1, ZBTB20-AS4, NNT-AS1, TRAF3IP2-AS1, and corresponding m5C regulators based CGGA dataset.

Supplementary Figure 2 **(A)** LASSO regression for 6 m5C-related lncRNAs based on TCGA dataset. **(B)** Differential expression of the 4 m5C-related lncRNAs in clinical subgroups (including WHO grade II or III, and age < =50 or >50 years). (**C**) K-M curves of the 4 m5C-related lncRNAs based on the CGGA dataset. (ns, non-significant, *p < 0.05, **p < 0.01, and ***p < 0.001).

Supplementary Figure 3 **(A)** The LAASO regression coefficients of the 4 selected m5C-related lncRNAs. **(B)** The distribution plots of the m5CrLS score and survival status in the CGGA dataset. **(C, D)** K-M curves of the m5CrLS-based stratification in multiple CGGA clinical subgroups. **(E)** ROC curves were used to evaluate the prediction ability of m5CrLS score, age, gender, grade, IDH, and 1p/19q status (1-, 3-, and 5-year).

Supplementary Figure 4 **(A)** Univariate Cox regression analysis of the TCGA and CGGA datasets. Heatmap of the relationship between the m5CrLS score and clinicopathological features in the **(B)** TCGA and **(C)** CGGA datasets (*p < 0.05, **p < 0.01, and ***p < 0.001).

Supplementary Figure 5 **(A)** The m5CrLS scores of clinicopathological subgroups in the CGGA dataset. **(B)** The principal component analysis (PCA) of high and low m5CrLS score, based on the expression of the 4 prognostic biomarkers, all genes, m5C-related lncRNAs, and m5C regulators.

Supplementary Figure 6 **(A)** The stromal score, immune score, estimate score, tumor purity of CGGA dataset. **(B)** Correlation of 5 immune cells from TIMER2.0 with the m5CrLS score. **(C)** Spearman correlation between six tumor infiltrating cells. **(D)** Chemokines and cytokines associated with dendritic cells were differentially expressed between high and low m5CrLS score in CGGA dataset. **(E)** Differences in 29 immune cells between high and low m5CrLS score patients of CGGA dataset. **(F)** The expression level of ICPs in the CGGA dataset. (ns, non-significant, *p < 0.05, **p < 0.01, and ***p < 0.001).

Supplementary Figure 7 K-M curves of **(A)** grade II and **(B)** grade III patients receiving radiotherapy or without radiotherapy based on TCGA dataset. K-M curves of **(C)** grade II and **(D)** grade III patients receiving radiotherapy or without radiotherapy based on CGGA dataset.
