# Supplementary material for: 5-Methylcytosine Related LncRNAs Reveal Immune Characteristics, Predict Prognosis and Oncology Treatment Outcome in Lower-Grade Gliomas
Source: Front Immunol. 2022 Mar 3;13:844778. doi: 10.3389/fimmu.2022.844778 (PMC8927645; doi:10.3389/fimmu.2022.844778)
Supplement: Supplementary file 1 [file DataSheet_1.docx]

**SUPPLEMENTARY TABLE 1 |** Top 10 in m5C network ranked by MCC method.

| Rank | Gene | Score |
| --- | --- | --- |
| 1 | TRDMT1 | 9 |
| 2 | DNMT3B | 8 |
| 2 | DNMT3A | 8 |
| 4 | TET2 | 6 |
| 4 | DNMT1 | 6 |
| 6 | NSUN7 | 5 |
| 6 | NSUN2 | 5 |
| 8 | NSUN5 | 4 |
| 8 | NOP2 | 4 |
| 10 | NSUN4 | 3 |
