# Supplementary material for: 5-Methylcytosine Related LncRNAs Reveal Immune Characteristics, Predict Prognosis and Oncology Treatment Outcome in Lower-Grade Gliomas
Source: Front Immunol. 2022 Mar 3;13:844778. doi: 10.3389/fimmu.2022.844778 (PMC8927645; doi:10.3389/fimmu.2022.844778)
Supplement: Supplementary file 2 [file DataSheet_2.docx]

**SUPPLEMENTARY TABLE 2 |** Univariate Cox analysis confirmed 107 m5C-related lncRNAs had prognostic value in TCGA dataset.

| Gene | HR | HR.95L | HR.95H | p-value |
| --- | --- | --- | --- | --- |
| AC110285.5 | 0.559138 | 0.33611 | 0.930159 | 0.025171 |
| AC012073.1 | 3.77287 | 2.702738 | 5.266715 | 6.09E-15 |
| AC021739.5 | 0.511442 | 0.395354 | 0.661616 | 3.31E-07 |
| AC013391.3 | 0.615232 | 0.510177 | 0.74192 | 3.68E-07 |
| AC097468.3 | 1.699874 | 1.058356 | 2.730246 | 0.028195 |
| AL122010.1 | 2.206497 | 1.396959 | 3.485164 | 0.00069 |
| AC015849.3 | 1.7433 | 1.229337 | 2.472141 | 0.001818 |
| AC078909.2 | 0.680512 | 0.482615 | 0.959556 | 0.028132 |
| AC017083.1 | 0.49069 | 0.290239 | 0.829582 | 0.007877 |
| AC007663.4 | 0.297299 | 0.170607 | 0.518071 | 1.86E-05 |
| AC073389.3 | 0.296944 | 0.197589 | 0.446259 | 5.15E-09 |
| LINC00609 | 0.629827 | 0.490889 | 0.808088 | 0.000277 |
| ARRDC1-AS1 | 0.630573 | 0.400023 | 0.993999 | 0.047046 |
| RBM26-AS1 | 0.552978 | 0.329905 | 0.926885 | 0.024571 |
| AL590666.2 | 0.732899 | 0.629899 | 0.852742 | 5.78E-05 |
| ATP2A1-AS1 | 1.437338 | 1.052308 | 1.963247 | 0.022581 |
| AL160006.1 | 1.804345 | 1.173556 | 2.774185 | 0.007163 |
| AC073842.2 | 2.423735 | 1.56742 | 3.747875 | 6.87E-05 |
| AC099850.3 | 2.013663 | 1.611866 | 2.515618 | 7.09E-10 |
| AL136368.1 | 0.601074 | 0.365437 | 0.988652 | 0.044972 |
| LINC00665 | 3.561648 | 2.357125 | 5.381701 | 1.63E-09 |
| LINC00265 | 1.809661 | 1.286927 | 2.544722 | 0.000649 |
| AC138207.2 | 0.568024 | 0.383562 | 0.841197 | 0.004756 |
| AC026471.4 | 0.671329 | 0.485834 | 0.927647 | 0.015729 |
| MIR181A2HG | 0.584607 | 0.395956 | 0.863139 | 0.006927 |
| AC129510.1 | 1.734668 | 1.166005 | 2.580669 | 0.006573 |
| LINC00928 | 0.617937 | 0.465723 | 0.8199 | 0.000849 |
| AC010761.1 | 1.692486 | 1.075991 | 2.662205 | 0.022793 |
| AC139795.2 | 2.367263 | 1.465954 | 3.82272 | 0.000425 |
| AP000757.1 | 0.390459 | 0.285138 | 0.534684 | 4.53E-09 |
| LINC01089 | 0.667753 | 0.517201 | 0.86213 | 0.001948 |
| DNAJC9-AS1 | 0.254915 | 0.14728 | 0.441212 | 1.04E-06 |
| TTC28-AS1 | 0.389759 | 0.23438 | 0.648143 | 0.000282 |
| AC005332.7 | 0.393646 | 0.224814 | 0.689269 | 0.001106 |
| ZNF32-AS1 | 0.526739 | 0.323856 | 0.856722 | 0.009792 |
| AF131215.5 | 0.682239 | 0.505752 | 0.920314 | 0.012291 |
| SNHG6 | 0.452237 | 0.323192 | 0.632807 | 3.67E-06 |
| CIRBP-AS1 | 2.040023 | 1.32703 | 3.136095 | 0.001156 |
| AL365330.1 | 1.453 | 1.017535 | 2.074827 | 0.039821 |
| Z84485.1 | 0.350171 | 0.198499 | 0.617736 | 0.000291 |
| AL390728.6 | 1.402638 | 1.028707 | 1.91249 | 0.032446 |
| AL353796.1 | 0.232905 | 0.141866 | 0.382366 | 8.37E-09 |
| AC004492.1 | 1.761631 | 1.065106 | 2.913648 | 0.027408 |
| NNT-AS1 | 0.542395 | 0.347974 | 0.845443 | 0.006906 |
| AC012510.1 | 1.652331 | 1.135424 | 2.404564 | 0.008704 |
| AC026401.3 | 2.07665 | 1.736392 | 2.483584 | 1.21E-15 |
| AC126118.1 | 0.596531 | 0.38897 | 0.91485 | 0.017891 |
| AL157392.3 | 0.103465 | 0.053198 | 0.201228 | 2.33E-11 |
| C8orf31 | 0.520815 | 0.31351 | 0.865199 | 0.011766 |
| AC026471.1 | 0.315862 | 0.164668 | 0.60588 | 0.000525 |
| AC022150.2 | 2.209059 | 1.427659 | 3.418141 | 0.000373 |
| GABPB1-AS1 | 0.706412 | 0.533372 | 0.935591 | 0.015335 |
| GDNF-AS1 | 0.302354 | 0.212009 | 0.431198 | 3.99E-11 |
| AC022364.1 | 1.464449 | 1.056931 | 2.029093 | 0.021863 |
| AC009283.1 | 0.689065 | 0.488507 | 0.971964 | 0.033838 |
| AC126407.1 | 0.57647 | 0.455727 | 0.729202 | 4.36E-06 |
| AL161935.3 | 1.251714 | 1.023177 | 1.531299 | 0.029057 |
| DICER1-AS1 | 0.32393 | 0.21965 | 0.477718 | 1.29E-08 |
| MAFG-DT | 0.663179 | 0.444206 | 0.990095 | 0.044575 |
| AP001767.3 | 0.58602 | 0.409137 | 0.839375 | 0.003556 |
| AL118505.1 | 0.56596 | 0.493128 | 0.649547 | 5.54E-16 |
| AL121890.5 | 0.482218 | 0.297963 | 0.780413 | 0.002984 |
| AL512625.2 | 0.617488 | 0.487587 | 0.781998 | 6.32E-05 |
| AC002310.1 | 0.323412 | 0.181364 | 0.576717 | 0.000131 |
| MIR9-3HG | 0.514932 | 0.445658 | 0.594974 | 2.18E-19 |
| LINC01719 | 0.399811 | 0.238835 | 0.669285 | 0.000488 |
| AC009955.4 | 0.596814 | 0.399831 | 0.890844 | 0.011552 |
| TPT1-AS1 | 0.466444 | 0.273304 | 0.796073 | 0.005171 |
| AC024601.1 | 0.188849 | 0.106099 | 0.336139 | 1.46E-08 |
| LINC00174 | 2.040206 | 1.38391 | 3.007738 | 0.000317 |
| AP001453.3 | 0.65422 | 0.435831 | 0.982041 | 0.040618 |
| AC009041.2 | 0.630227 | 0.555715 | 0.714732 | 6.41E-13 |
| AL359881.3 | 0.380633 | 0.218055 | 0.664429 | 0.000678 |
| AL450384.2 | 0.531979 | 0.358411 | 0.789602 | 0.001734 |
| AL158212.2 | 0.379049 | 0.211533 | 0.679221 | 0.001115 |
| AC095057.3 | 0.521944 | 0.357933 | 0.761107 | 0.000729 |
| MIR325HG | 0.566875 | 0.411895 | 0.780168 | 0.000495 |
| RGMB-AS1 | 0.357486 | 0.225032 | 0.5679 | 1.33E-05 |
| AC021739.3 | 0.558626 | 0.439469 | 0.710092 | 1.97E-06 |
| ZEB1-AS1 | 0.579462 | 0.36932 | 0.909173 | 0.017583 |
| AL139011.1 | 1.929804 | 1.1285 | 3.300084 | 0.016325 |
| AL035461.2 | 2.085049 | 1.585485 | 2.742017 | 1.46E-07 |
| SNHG11 | 1.485088 | 1.145723 | 1.924975 | 0.002811 |
| AL138960.1 | 0.538522 | 0.396712 | 0.731024 | 7.21E-05 |
| AC092718.4 | 2.392088 | 1.926303 | 2.970502 | 2.94E-15 |
| AC125807.2 | 1.310822 | 1.021091 | 1.682763 | 0.033692 |
| AL391425.1 | 0.481656 | 0.297927 | 0.778689 | 0.002877 |
| AL033384.2 | 0.569751 | 0.368489 | 0.880938 | 0.011403 |
| AL159169.2 | 0.503897 | 0.353717 | 0.71784 | 0.000147 |
| AC009227.1 | 0.265926 | 0.183878 | 0.384586 | 1.97E-12 |
| AL158055.1 | 0.299739 | 0.176814 | 0.508123 | 7.68E-06 |
| ZIM2-AS1 | 2.430611 | 1.761305 | 3.354259 | 6.50E-08 |
| AC010536.3 | 0.315624 | 0.185075 | 0.538261 | 2.29E-05 |
| AC244517.7 | 0.719015 | 0.573652 | 0.901213 | 0.004202 |
| GLYCTK-AS1 | 0.460454 | 0.322518 | 0.657384 | 1.96E-05 |
| AP006623.1 | 0.528179 | 0.322949 | 0.863831 | 0.010986 |
| AC009948.1 | 1.782623 | 1.16822 | 2.720161 | 0.007339 |
| ZBTB20-AS4 | 0.253652 | 0.162979 | 0.394769 | 1.21E-09 |
| AL117332.1 | 4.670214 | 2.865794 | 7.610769 | 6.19E-10 |
| AL021368.3 | 0.585921 | 0.416418 | 0.82442 | 0.002154 |
| TRAF3IP2-AS1 | 0.378127 | 0.244873 | 0.583897 | 1.15E-05 |
| PTOV1-AS2 | 1.4991 | 1.194062 | 1.882065 | 0.000487 |
| SNHG1 | 0.647906 | 0.51267 | 0.818816 | 0.00028 |
| AL391807.1 | 0.678502 | 0.550222 | 0.836689 | 0.000286 |
| AC116914.2 | 1.720967 | 1.145677 | 2.585134 | 0.008922 |
| ZNF32-AS2 | 0.644735 | 0.422906 | 0.98292 | 0.041347 |
| AC131009.3 | 1.910119 | 1.259771 | 2.896204 | 0.002309 |
