# Supplementary material for: 5-Methylcytosine Related LncRNAs Reveal Immune Characteristics, Predict Prognosis and Oncology Treatment Outcome in Lower-Grade Gliomas
Source: Front Immunol. 2022 Mar 3;13:844778. doi: 10.3389/fimmu.2022.844778 (PMC8927645; doi:10.3389/fimmu.2022.844778)
Supplement: Supplementary file 3 [file DataSheet_3.docx]

**SUPPLEMENTARY TABLE 3 |** Univariate Cox analysis confirmed 46 m5C-related lncRNAs had prognostic value in CGGA dataset.

| Gene | HR | HR.95L | HR.95H | p*-*value |
| --- | --- | --- | --- | --- |
| AC005253.2 | 1.329487 | 1.06719 | 1.656252 | 0.011088 |
| AC007383.3 | 1.266281 | 1.04063 | 1.540863 | 0.018389 |
| AC007620.3 | 1.253293 | 1.132019 | 1.387558 | 1.37E-05 |
| AC018730.1 | 1.170457 | 1.041242 | 1.315707 | 0.008362 |
| AC024560.3 | 0.856353 | 0.743372 | 0.986505 | 0.0317 |
| AC090425.1 | 0.786051 | 0.682053 | 0.905905 | 0.000885 |
| AC096677.1 | 1.203169 | 1.010573 | 1.432469 | 0.037697 |
| AC117395.1 | 1.322867 | 1.148062 | 1.524287 | 0.000109 |
| AC122129.1 | 0.751034 | 0.607023 | 0.92921 | 0.008391 |
| AGAP2-AS1 | 1.430912 | 1.308854 | 1.564352 | 3.36E-15 |
| ANKRD10-IT1 | 1.237818 | 1.063553 | 1.440638 | 0.005854 |
| CIRBP-AS1 | 1.368492 | 1.158476 | 1.616582 | 0.000224 |
| COX10-AS1 | 1.654482 | 1.336048 | 2.048813 | 3.91E-06 |
| DCTN1-AS1 | 0.781947 | 0.662381 | 0.923097 | 0.003672 |
| EMX2OS | 0.7364 | 0.661708 | 0.819524 | 2.05E-08 |
| FBXL19-AS1 | 1.459059 | 1.152331 | 1.847433 | 0.001704 |
| GDNF-AS1 | 0.705483 | 0.624788 | 0.796599 | 1.81E-08 |
| HCG18 | 0.789982 | 0.656751 | 0.95024 | 0.012364 |
| INE1 | 1.214239 | 1.028128 | 1.434039 | 0.022211 |
| LENG8-AS1 | 1.330866 | 1.120484 | 1.580749 | 0.001131 |
| LIFR-AS1 | 0.682064 | 0.54538 | 0.853005 | 0.000798 |
| LINC00092 | 1.307693 | 1.155489 | 1.479944 | 2.15E-05 |
| LINC00115 | 1.601745 | 1.30621 | 1.964145 | 5.99E-06 |
| LINC00265 | 1.567666 | 1.297131 | 1.894624 | 3.29E-06 |
| LINC00461 | 0.873098 | 0.777407 | 0.980568 | 0.021947 |
| LINC00515 | 0.508653 | 0.42099 | 0.614571 | 2.48E-12 |
| LINC00662 | 1.947519 | 1.647248 | 2.302525 | 6.10E-15 |
| LINC01001 | 1.176293 | 1.021048 | 1.355141 | 0.024551 |
| LINC01004 | 1.236014 | 1.048465 | 1.457112 | 0.011615 |
| LOXL1-AS1 | 1.779386 | 1.493233 | 2.120376 | 1.18E-10 |
| MCM3AP-AS1 | 0.668928 | 0.545169 | 0.82078 | 0.000117 |
| MIR497HG | 0.611648 | 0.536586 | 0.69721 | 1.85E-13 |
| NEAT1 | 1.304464 | 1.204715 | 1.412473 | 5.81E-11 |
| NNT-AS1 | 0.717223 | 0.595948 | 0.863177 | 0.000437 |
| PAXIP1-AS2 | 1.862771 | 1.53728 | 2.257179 | 2.17E-10 |
| RAMP2-AS1 | 0.820606 | 0.702018 | 0.959226 | 0.013039 |
| RNF139-AS1 | 1.455218 | 1.126078 | 1.880563 | 0.004136 |
| SH3BP5-AS1 | 1.491575 | 1.270238 | 1.751481 | 1.07E-06 |
| SNHG16 | 1.557248 | 1.28265 | 1.890634 | 7.64E-06 |
| ST7-AS1 | 1.33787 | 1.121266 | 1.596316 | 0.001237 |
| STXBP5-AS1 | 0.447499 | 0.345102 | 0.58028 | 1.32E-09 |
| THAP9-AS1 | 1.487187 | 1.261479 | 1.753279 | 2.29E-06 |
| TMCC1-AS1 | 0.654638 | 0.543351 | 0.78872 | 8.33E-06 |
| TMEM254-AS1 | 0.524406 | 0.416031 | 0.661012 | 4.63E-08 |
| TRAF3IP2-AS1 | 0.659377 | 0.530529 | 0.819519 | 0.000174 |
| ZBTB20-AS4 | 0.621963 | 0.487052 | 0.794242 | 0.000141 |
