# Supplementary material for: 5-Methylcytosine Related LncRNAs Reveal Immune Characteristics, Predict Prognosis and Oncology Treatment Outcome in Lower-Grade Gliomas
Source: Front Immunol. 2022 Mar 3;13:844778. doi: 10.3389/fimmu.2022.844778 (PMC8927645; doi:10.3389/fimmu.2022.844778)
Supplement: Supplementary file 4 [file DataSheet_4.docx]

**SUPPLEMENTARY TABLE 4 |** The correlations between m5C regulators and lncRNAs based on the CGGA dataset.

| lncRNAs | m5C | cor | p-value | Regulation |
| --- | --- | --- | --- | --- |
| CIRBP-AS1 | DNMT3B | 0.5039 | 2.16E-34 | postive |
| GDNF-AS1 | NSUN6 | 0.5273 | 4.71E-38 | postive |
| LINC00265 | DNMT3B | 0.5116 | 1.48E-35 | postive |
| LINC00265 | NOP2 | 0.5315 | 9.64E-39 | postive |
| ZBTB20-AS4 | NSUN3 | 0.5140 | 6.16E-36 | postive |
| ZBTB20-AS4 | TET2 | 0.5858 | 1.38E-48 | postive |
| NNT-AS1 | NSUN3 | 0.5809 | 1.26E-47 | postive |
| NNT-AS1 | TET2 | 0.6095 | 1.72E-53 | postive |
