# Supplementary material for: 5-Methylcytosine Related LncRNAs Reveal Immune Characteristics, Predict Prognosis and Oncology Treatment Outcome in Lower-Grade Gliomas
Source: Front Immunol. 2022 Mar 3;13:844778. doi: 10.3389/fimmu.2022.844778 (PMC8927645; doi:10.3389/fimmu.2022.844778)
Supplement: Supplementary file 5 [file DataSheet_5.docx]

**SUPPLEMENTARY TABLE 5 |** 649 DEGs in high and low m5CrLS score groups base on TCGA dataset.

| Gene | lowMean | highMean | logFC | p-value | FDR |
| --- | --- | --- | --- | --- | --- |
| EVC | 0.5627 | 1.4616 | 1.3772 | 4.74E-14 | 2.31E-13 |
| PPIC | 2.1305 | 4.5593 | 1.0977 | 2.73E-08 | 6.83E-08 |
| MYT1 | 12.5547 | 5.7023 | -1.1386 | 1.25E-34 | 1.62E-32 |
| PLAU | 1.5863 | 5.8694 | 1.8876 | 4.57E-08 | 1.11E-07 |
| PKIB | 0.7628 | 2.3129 | 1.6002 | 7.45E-11 | 2.48E-10 |
| KLHDC8A | 8.7012 | 18.3791 | 1.0788 | 1.14E-02 | 1.50E-02 |
| ITGA3 | 2.6715 | 5.7886 | 1.1156 | 2.49E-11 | 8.74E-11 |
| JCHAIN | 0.2563 | 2.0783 | 3.0192 | 5.75E-03 | 7.84E-03 |
| TMEM176B | 12.0603 | 29.6567 | 1.2981 | 4.53E-23 | 7.53E-22 |
| NAMPT | 5.8636 | 11.9474 | 1.0268 | 4.63E-06 | 9.15E-06 |
| DKK1 | 0.4693 | 1.9460 | 2.0520 | 2.14E-02 | 2.71E-02 |
| GMPR | 4.4482 | 14.2530 | 1.6800 | 3.18E-27 | 9.98E-26 |
| BMP2 | 27.8743 | 10.4317 | -1.4180 | 4.29E-46 | 9.45E-43 |
| RGN | 2.0456 | 5.7849 | 1.4998 | 1.78E-19 | 1.73E-18 |
| ICAM1 | 2.2355 | 5.0623 | 1.1792 | 5.27E-10 | 1.59E-09 |
| PIPOX | 3.6527 | 10.0406 | 1.4588 | 1.31E-14 | 6.83E-14 |
| LHFPL3 | 61.4713 | 28.2572 | -1.1213 | 5.40E-32 | 4.08E-30 |
| FGFBP2 | 0.6553 | 1.8030 | 1.4602 | 7.31E-12 | 2.74E-11 |
| SLC2A10 | 1.2191 | 3.4441 | 1.4983 | 9.89E-24 | 1.78E-22 |
| TRIP6 | 10.6162 | 24.6115 | 1.2131 | 1.27E-28 | 4.93E-27 |
| SERTM1 | 0.6274 | 1.4728 | 1.2312 | 8.03E-03 | 1.08E-02 |
| AC009902.2 | 0.7106 | 1.5373 | 1.1133 | 3.39E-12 | 1.33E-11 |
| IGHG4 | 0.2619 | 1.7826 | 2.7671 | 4.80E-03 | 6.62E-03 |
| MAOB | 18.4978 | 67.1705 | 1.8605 | 8.96E-24 | 1.62E-22 |
| PRODH | 0.9517 | 1.9703 | 1.0499 | 8.12E-10 | 2.40E-09 |
| EFEMP1 | 25.7987 | 52.5697 | 1.0269 | 2.96E-09 | 8.22E-09 |
| FBXO17 | 0.5537 | 2.5150 | 2.1832 | 6.01E-24 | 1.10E-22 |
| AL049749.1 | 4.1069 | 1.5136 | -1.4400 | 9.35E-21 | 1.09E-19 |
| TTR | 0.0223 | 209.7633 | 13.1968 | 2.13E-05 | 3.93E-05 |
| BCAT1 | 1.2979 | 4.2566 | 1.7136 | 2.25E-14 | 1.14E-13 |
| KLRC2 | 15.5769 | 6.4516 | -1.2717 | 1.38E-30 | 7.93E-29 |
| FAM50B | 2.4857 | 5.9013 | 1.2474 | 3.47E-21 | 4.28E-20 |
| THNSL2 | 0.7645 | 1.7688 | 1.2101 | 2.80E-11 | 9.76E-11 |
| SAMD9L | 2.1834 | 4.5672 | 1.0647 | 2.32E-11 | 8.17E-11 |
| IGHG2 | 0.9309 | 4.4927 | 2.2709 | 4.18E-08 | 1.02E-07 |
| CXCL14 | 8.6104 | 29.5362 | 1.7783 | 2.20E-09 | 6.20E-09 |
| FKBP5 | 4.9338 | 10.2123 | 1.0495 | 2.29E-12 | 9.16E-12 |
| TRBC2 | 0.5912 | 1.4739 | 1.3179 | 3.44E-12 | 1.35E-11 |
| SPRY4 | 3.2821 | 8.4198 | 1.3591 | 1.66E-05 | 3.09E-05 |
| RYR3 | 0.5296 | 1.6126 | 1.6064 | 5.78E-16 | 3.56E-15 |
| GDF15 | 0.3354 | 2.0377 | 2.6030 | 4.99E-10 | 1.51E-09 |
| TMEM221 | 0.6986 | 1.7571 | 1.3307 | 8.06E-21 | 9.50E-20 |
| PLAUR | 0.8545 | 2.3619 | 1.4668 | 5.34E-21 | 6.47E-20 |
| RUNX1 | 0.6504 | 1.4683 | 1.1747 | 3.53E-11 | 1.22E-10 |
| AC098587.1 | 1.6980 | 0.7493 | -1.1801 | 1.66E-25 | 3.94E-24 |
| TSTD1 | 1.0893 | 5.4051 | 2.3110 | 2.67E-22 | 3.89E-21 |
| TACR1 | 3.0622 | 1.0678 | -1.5200 | 1.32E-22 | 2.03E-21 |
| MOXD1 | 0.4861 | 7.2989 | 3.9083 | 1.00E-23 | 1.79E-22 |
| XKR7 | 2.3927 | 0.7571 | -1.6601 | 2.30E-20 | 2.52E-19 |
| LINC02440 | 4.3866 | 1.6773 | -1.3870 | 7.89E-27 | 2.29E-25 |
| SYNPO | 5.3250 | 11.8254 | 1.1510 | 1.06E-12 | 4.39E-12 |
| DLX1 | 0.8661 | 1.9542 | 1.1740 | 5.90E-05 | 1.03E-04 |
| LRRN4CL | 1.0178 | 2.3449 | 1.2041 | 4.70E-06 | 9.28E-06 |
| C4orf47 | 0.9471 | 2.0606 | 1.1215 | 2.45E-08 | 6.15E-08 |
| SRPX2 | 1.0431 | 3.8617 | 1.8883 | 1.92E-09 | 5.44E-09 |
| DBNDD1 | 3.7236 | 7.8891 | 1.0831 | 1.56E-21 | 2.05E-20 |
| CCL2 | 7.1450 | 18.3730 | 1.3626 | 1.51E-09 | 4.35E-09 |
| AK4 | 4.2895 | 8.7203 | 1.0236 | 3.01E-18 | 2.50E-17 |
| C1orf194 | 2.1044 | 5.3453 | 1.3449 | 4.80E-08 | 1.16E-07 |
| COL5A3 | 5.4448 | 10.9409 | 1.0068 | 6.81E-19 | 6.11E-18 |
| FCGBP | 5.3967 | 12.5692 | 1.2197 | 5.61E-07 | 1.22E-06 |
| HSPB1P1 | 0.8709 | 1.8069 | 1.0529 | 1.35E-14 | 7.01E-14 |
| RNU6-277P | 3.0897 | 1.3589 | -1.1851 | 2.65E-28 | 9.74E-27 |
| SKAP2 | 2.7136 | 6.7172 | 1.3076 | 1.18E-23 | 2.10E-22 |
| AC010424.1 | 1.0488 | 3.1111 | 1.5687 | 2.85E-17 | 2.10E-16 |
| OAS1 | 2.5669 | 5.2666 | 1.0369 | 1.90E-10 | 6.04E-10 |
| MMD2 | 22.2379 | 10.0587 | -1.1446 | 1.15E-16 | 7.74E-16 |
| GRIN3A | 2.8655 | 1.1800 | -1.2800 | 1.82E-14 | 9.33E-14 |
| SAPCD2 | 24.5973 | 11.6220 | -1.0816 | 2.43E-27 | 7.72E-26 |
| RARRES2 | 1.0182 | 10.6499 | 3.3868 | 2.89E-28 | 1.05E-26 |
| AMZ1 | 3.2477 | 1.5471 | -1.0699 | 3.12E-22 | 4.49E-21 |
| ECM2 | 2.6385 | 6.5073 | 1.3023 | 4.72E-13 | 2.05E-12 |
| CP | 1.7253 | 5.3270 | 1.6265 | 1.47E-13 | 6.78E-13 |
| MT1L | 2.9316 | 6.8974 | 1.2344 | 1.06E-17 | 8.26E-17 |
| MIR4666A | 1.6704 | 0.7946 | -1.0719 | 2.34E-20 | 2.56E-19 |
| SLC7A10 | 0.4716 | 2.6286 | 2.4787 | 6.70E-10 | 2.00E-09 |
| S100A3 | 1.4587 | 2.9639 | 1.0228 | 3.25E-12 | 1.28E-11 |
| DES | 0.4829 | 2.2420 | 2.2150 | 1.82E-17 | 1.36E-16 |
| HLA-DQB2 | 1.0192 | 2.4682 | 1.2759 | 2.85E-09 | 7.94E-09 |
| CXCL1 | 0.8252 | 1.9452 | 1.2371 | 7.79E-03 | 1.05E-02 |
| FKBP9 | 4.8071 | 12.5701 | 1.3868 | 2.44E-28 | 9.11E-27 |
| SLC26A4-AS1 | 0.9191 | 1.9781 | 1.1059 | 1.69E-03 | 2.48E-03 |
| SNTA1 | 25.8616 | 53.4663 | 1.0478 | 1.01E-23 | 1.80E-22 |
| SPEF1 | 0.9542 | 3.6516 | 1.9362 | 2.66E-19 | 2.54E-18 |
| ARX | 0.9470 | 2.2096 | 1.2223 | 1.02E-06 | 2.17E-06 |
| LSP1 | 0.9334 | 2.5307 | 1.4389 | 4.38E-16 | 2.74E-15 |
| ECHDC2 | 0.6935 | 1.8788 | 1.4379 | 2.12E-20 | 2.34E-19 |
| SEC61G | 14.8324 | 66.9442 | 2.1742 | 8.72E-05 | 1.49E-04 |
| B3GNT7 | 0.4033 | 1.9075 | 2.2418 | 3.67E-20 | 3.89E-19 |
| PSRC1 | 8.8934 | 21.6516 | 1.2837 | 9.68E-31 | 5.74E-29 |
| NIPAL2 | 0.8136 | 1.8179 | 1.1600 | 6.69E-14 | 3.20E-13 |
| PLAT | 1.6798 | 8.9043 | 2.4063 | 1.54E-22 | 2.34E-21 |
| AL513217.1 | 2.6209 | 0.9356 | -1.4862 | 6.28E-21 | 7.51E-20 |
| IGKV1-5 | 0.5144 | 2.1799 | 2.0833 | 4.40E-05 | 7.81E-05 |
| MMP24 | 8.6308 | 4.2410 | -1.0251 | 1.10E-19 | 1.10E-18 |
| TMBIM1 | 8.8650 | 18.9202 | 1.0937 | 8.77E-32 | 6.27E-30 |
| WWTR1 | 5.9365 | 11.9614 | 1.0107 | 6.82E-14 | 3.26E-13 |
| HLA-DQA2 | 3.0086 | 7.2834 | 1.2755 | 7.89E-05 | 1.36E-04 |
| AEBP1 | 14.3583 | 55.2039 | 1.9429 | 1.90E-20 | 2.11E-19 |
| ARAP3 | 1.7883 | 3.7061 | 1.0514 | 1.35E-06 | 2.83E-06 |
| LATS2 | 0.8735 | 1.8429 | 1.0771 | 1.63E-16 | 1.07E-15 |
| PTX3 | 0.8410 | 2.7195 | 1.6931 | 7.14E-04 | 1.09E-03 |
| MYBPC1 | 2.4767 | 5.4531 | 1.1386 | 7.01E-15 | 3.77E-14 |
| SEL1L3 | 1.1999 | 4.7079 | 1.9721 | 1.51E-14 | 7.78E-14 |
| PALM3 | 0.7989 | 1.6313 | 1.0300 | 4.07E-15 | 2.25E-14 |
| ISLR | 3.5638 | 8.0125 | 1.1688 | 1.55E-09 | 4.45E-09 |
| VWC2L | 1.5765 | 0.7245 | -1.1217 | 6.59E-09 | 1.77E-08 |
| OXTR | 1.8147 | 5.2016 | 1.5193 | 2.27E-12 | 9.09E-12 |
| DMRTA2 | 0.1375 | 1.9858 | 3.8520 | 6.15E-11 | 2.07E-10 |
| ANXA2 | 3.9540 | 12.1315 | 1.6174 | 7.00E-19 | 6.26E-18 |
| KCNK3 | 5.3704 | 2.1055 | -1.3508 | 1.34E-21 | 1.77E-20 |
| SFRP4 | 1.6249 | 6.9296 | 2.0924 | 2.13E-18 | 1.80E-17 |
| CAV2 | 1.0888 | 2.3305 | 1.0980 | 4.82E-09 | 1.31E-08 |
| AIFM3 | 2.2293 | 5.4650 | 1.2936 | 6.60E-08 | 1.58E-07 |
| KLHL26 | 1.4908 | 4.2539 | 1.5127 | 5.22E-29 | 2.19E-27 |
| CRNDE | 0.9266 | 2.4574 | 1.4071 | 4.70E-10 | 1.43E-09 |
| CITED4 | 1.6089 | 3.3798 | 1.0708 | 3.08E-18 | 2.55E-17 |
| DPYD | 1.1629 | 2.8469 | 1.2917 | 1.85E-12 | 7.48E-12 |
| SLC44A3 | 2.1244 | 4.3105 | 1.0208 | 1.00E-25 | 2.48E-24 |
| THBS4 | 5.0540 | 14.4041 | 1.5110 | 2.59E-04 | 4.17E-04 |
| ISG15 | 34.9071 | 70.0147 | 1.0041 | 1.42E-09 | 4.09E-09 |
| IGHM | 0.4421 | 4.6582 | 3.3972 | 9.93E-06 | 1.89E-05 |
| C2CD4D-AS1 | 0.8755 | 1.9869 | 1.1823 | 6.81E-08 | 1.62E-07 |
| C21orf62 | 2.1157 | 5.1150 | 1.2736 | 1.14E-10 | 3.75E-10 |
| ST8SIA5 | 0.7818 | 2.1823 | 1.4810 | 5.38E-06 | 1.05E-05 |
| HSPA6 | 1.2553 | 5.5578 | 2.1465 | 5.90E-12 | 2.24E-11 |
| POSTN | 0.1358 | 10.5504 | 6.2796 | 3.87E-12 | 1.50E-11 |
| PIFO | 1.7284 | 5.7595 | 1.7365 | 6.47E-25 | 1.39E-23 |
| PPL | 0.5064 | 1.6597 | 1.7125 | 2.69E-10 | 8.41E-10 |
| ANKRD35 | 1.9330 | 4.3347 | 1.1651 | 3.71E-25 | 8.34E-24 |
| RAMP3 | 5.6712 | 12.7343 | 1.1670 | 3.43E-11 | 1.19E-10 |
| COL4A1 | 6.4799 | 18.4849 | 1.5123 | 4.96E-03 | 6.82E-03 |
| TOX3 | 7.1589 | 3.4032 | -1.0728 | 6.11E-36 | 1.09E-33 |
| CXCL10 | 0.9836 | 5.1613 | 2.3916 | 8.48E-10 | 2.50E-09 |
| TIMP1 | 8.1503 | 61.6335 | 2.9188 | 3.04E-23 | 5.15E-22 |
| MRGPRF | 1.1188 | 2.3020 | 1.0409 | 1.16E-09 | 3.38E-09 |
| GSDMD | 2.0153 | 4.2971 | 1.0924 | 4.26E-19 | 3.94E-18 |
| AL590666.2 | 15.4971 | 6.0748 | -1.3511 | 2.19E-36 | 4.25E-34 |
| CXCL8 | 1.0287 | 2.9350 | 1.5126 | 3.40E-03 | 4.78E-03 |
| PCP4 | 4.0495 | 9.4208 | 1.2181 | 4.93E-06 | 9.69E-06 |
| LHX5 | 2.3126 | 0.5851 | -1.9827 | 2.49E-16 | 1.60E-15 |
| MARCHF4 | 4.9662 | 1.9914 | -1.3183 | 2.02E-12 | 8.12E-12 |
| ACAA2 | 3.4667 | 7.0144 | 1.0168 | 9.41E-30 | 4.59E-28 |
| LGALS3 | 7.1080 | 20.2184 | 1.5082 | 1.74E-19 | 1.70E-18 |
| SERTAD4-AS1 | 0.7621 | 1.6004 | 1.0704 | 6.49E-12 | 2.44E-11 |
| CRTAC1 | 29.9684 | 13.1423 | -1.1892 | 3.36E-32 | 2.70E-30 |
| HOXD9 | 0.5901 | 1.5454 | 1.3890 | 2.25E-03 | 3.24E-03 |
| CPNE6 | 2.5450 | 5.9639 | 1.2286 | 1.62E-04 | 2.69E-04 |
| LHX5-AS1 | 6.2298 | 1.5804 | -1.9789 | 1.33E-17 | 1.02E-16 |
| APLNR | 28.2809 | 64.6640 | 1.1931 | 9.54E-11 | 3.15E-10 |
| VWA3A | 0.7172 | 1.6019 | 1.1593 | 1.05E-02 | 1.38E-02 |
| AGAP2-AS1 | 0.6652 | 5.0362 | 2.9205 | 2.22E-17 | 1.65E-16 |
| CPNE8 | 0.6040 | 1.4865 | 1.2993 | 3.59E-11 | 1.24E-10 |
| CFH | 1.1407 | 2.6995 | 1.2427 | 1.26E-07 | 2.94E-07 |
| RGS14 | 0.8685 | 2.1527 | 1.3095 | 1.78E-17 | 1.34E-16 |
| SERPING1 | 8.7615 | 32.6812 | 1.8992 | 9.97E-28 | 3.42E-26 |
| LTBP1 | 3.5941 | 7.4815 | 1.0577 | 1.47E-17 | 1.12E-16 |
| ASS1 | 3.4023 | 6.8187 | 1.0030 | 4.17E-14 | 2.04E-13 |
| COL5A2 | 1.8597 | 6.1220 | 1.7189 | 2.94E-10 | 9.15E-10 |
| COX7A1 | 4.7669 | 10.9922 | 1.2054 | 1.99E-07 | 4.53E-07 |
| RSPO2 | 0.9172 | 2.0516 | 1.1613 | 1.68E-04 | 2.77E-04 |
| ERICH3 | 0.7818 | 1.8799 | 1.2658 | 1.07E-05 | 2.03E-05 |
| COL9A3 | 3.2539 | 9.9126 | 1.6071 | 1.99E-16 | 1.30E-15 |
| LINC00928 | 2.4000 | 1.1116 | -1.1103 | 3.20E-28 | 1.16E-26 |
| AC012558.1 | 1.9188 | 0.7783 | -1.3018 | 1.08E-30 | 6.34E-29 |
| RIBC1 | 0.8008 | 1.6307 | 1.0260 | 3.89E-05 | 6.94E-05 |
| F2RL1 | 1.3001 | 2.6853 | 1.0464 | 3.48E-09 | 9.62E-09 |
| PDGFA | 4.3711 | 12.2213 | 1.4833 | 1.61E-20 | 1.81E-19 |
| EMP1 | 9.3258 | 21.4893 | 1.2043 | 6.30E-15 | 3.41E-14 |
| MMP14 | 9.7272 | 21.3505 | 1.1342 | 3.02E-13 | 1.34E-12 |
| GBP1 | 3.0689 | 10.9240 | 1.8317 | 3.83E-17 | 2.77E-16 |
| NPTX2 | 5.3034 | 13.6104 | 1.3597 | 2.03E-07 | 4.64E-07 |
| APOL1 | 2.5046 | 5.7606 | 1.2017 | 4.53E-09 | 1.23E-08 |
| C16orf89 | 2.5575 | 7.0613 | 1.4652 | 1.79E-24 | 3.60E-23 |
| PCDHGA3 | 2.2502 | 1.1062 | -1.0245 | 1.54E-02 | 1.99E-02 |
| AP000757.1 | 3.0303 | 1.3798 | -1.1350 | 5.70E-36 | 1.03E-33 |
| VIM | 108.1163 | 252.1393 | 1.2216 | 1.19E-18 | 1.03E-17 |
| TPPP3 | 9.1476 | 30.4929 | 1.7370 | 5.20E-19 | 4.75E-18 |
| NPNT | 1.4581 | 6.2794 | 2.1065 | 4.67E-08 | 1.13E-07 |
| LIF | 0.2837 | 1.7721 | 2.6431 | 7.24E-06 | 1.40E-05 |
| COL1A1 | 2.0375 | 12.7467 | 2.6452 | 2.37E-04 | 3.84E-04 |
| CRABP1 | 0.7477 | 2.6184 | 1.8081 | 4.28E-06 | 8.48E-06 |
| DLL3 | 131.0737 | 61.1436 | -1.1001 | 2.90E-26 | 7.76E-25 |
| CD58 | 1.5868 | 3.9481 | 1.3150 | 4.80E-24 | 8.93E-23 |
| TP73-AS1 | 1.0513 | 3.1776 | 1.5957 | 1.76E-25 | 4.15E-24 |
| NR4A3 | 0.9420 | 1.9799 | 1.0716 | 7.67E-05 | 1.32E-04 |
| LINC00237 | 5.4240 | 2.5354 | -1.0972 | 1.32E-37 | 3.56E-35 |
| AL136114.1 | 3.6878 | 1.5755 | -1.2269 | 3.01E-18 | 2.50E-17 |
| IGLV3-21 | 0.1330 | 1.9368 | 3.8640 | 7.33E-04 | 1.12E-03 |
| IGFBP5 | 38.4355 | 78.2627 | 1.0259 | 1.72E-17 | 1.30E-16 |
| MCUB | 1.0398 | 3.2438 | 1.6414 | 1.86E-23 | 3.22E-22 |
| FOLR1 | 0.6954 | 2.4767 | 1.8324 | 2.44E-08 | 6.13E-08 |
| VASN | 0.8160 | 4.9167 | 2.5910 | 1.34E-35 | 2.16E-33 |
| ACTN1 | 5.3612 | 13.4916 | 1.3314 | 2.49E-16 | 1.60E-15 |
| S100A13 | 5.7082 | 14.4649 | 1.3415 | 1.60E-27 | 5.32E-26 |
| CHST6 | 1.3717 | 3.9846 | 1.5385 | 1.73E-27 | 5.70E-26 |
| HSPB6 | 5.9100 | 22.7715 | 1.9460 | 2.08E-29 | 9.53E-28 |
| PYGM | 2.2579 | 6.8203 | 1.5948 | 4.53E-13 | 1.97E-12 |
| TNFRSF1A | 11.1934 | 22.3881 | 1.0001 | 2.25E-24 | 4.43E-23 |
| NGFR | 2.8173 | 8.5731 | 1.6055 | 5.71E-04 | 8.83E-04 |
| PPP1R1B | 10.7353 | 25.8433 | 1.2674 | 4.62E-12 | 1.77E-11 |
| RBP1 | 1.9753 | 12.2609 | 2.6339 | 2.92E-18 | 2.43E-17 |
| TUBB6 | 3.3484 | 7.6128 | 1.1850 | 9.40E-14 | 4.43E-13 |
| PCDHGA1 | 2.1781 | 1.0553 | -1.0454 | 1.62E-09 | 4.64E-09 |
| CYTL1 | 3.2884 | 6.6948 | 1.0256 | 2.74E-12 | 1.09E-11 |
| AL022313.4 | 11.4115 | 5.6552 | -1.0128 | 8.38E-16 | 5.05E-15 |
| GPR3 | 0.7351 | 1.7016 | 1.2108 | 1.70E-10 | 5.45E-10 |
| C1S | 6.4702 | 17.3010 | 1.4190 | 7.53E-13 | 3.19E-12 |
| OCIAD2 | 1.3704 | 5.9726 | 2.1238 | 1.91E-21 | 2.45E-20 |
| NMNAT3 | 1.0749 | 2.3042 | 1.1000 | 7.62E-20 | 7.75E-19 |
| HOTAIRM1 | 0.4380 | 2.6319 | 2.5872 | 7.04E-10 | 2.09E-09 |
| APOBEC3C | 3.1260 | 6.4444 | 1.0437 | 1.89E-13 | 8.62E-13 |
| MFAP4 | 6.3633 | 13.9520 | 1.1326 | 4.25E-11 | 1.45E-10 |
| CYB561 | 2.3932 | 4.9903 | 1.0602 | 1.07E-26 | 3.08E-25 |
| FERMT1 | 23.5955 | 9.2646 | -1.3487 | 4.32E-43 | 5.91E-40 |
| SERPINE1 | 4.5271 | 17.4952 | 1.9503 | 1.85E-15 | 1.07E-14 |
| LINC02587 | 0.1096 | 2.7264 | 4.6366 | 1.50E-06 | 3.13E-06 |
| MT1E | 24.0335 | 59.6785 | 1.3122 | 3.91E-15 | 2.17E-14 |
| ZDHHC8P1 | 0.6335 | 1.9169 | 1.5974 | 1.18E-12 | 4.91E-12 |
| METTL7B | 4.8592 | 26.8371 | 2.4654 | 8.16E-20 | 8.27E-19 |
| TEKT1 | 0.3496 | 1.7454 | 2.3197 | 1.04E-10 | 3.41E-10 |
| MET | 0.6880 | 2.8620 | 2.0566 | 8.49E-12 | 3.16E-11 |
| CSMD3 | 3.0921 | 1.1600 | -1.4145 | 1.78E-41 | 1.39E-38 |
| IGLV2-14 | 0.2370 | 1.7983 | 2.9239 | 4.06E-07 | 8.99E-07 |
| FABP5 | 1.1836 | 6.6252 | 2.4848 | 1.85E-26 | 5.11E-25 |
| HAMP | 1.1071 | 2.4342 | 1.1367 | 1.00E-15 | 5.98E-15 |
| HCP5 | 1.6739 | 3.3489 | 1.0004 | 1.44E-09 | 4.14E-09 |
| WIF1 | 1.5215 | 4.5927 | 1.5938 | 7.01E-03 | 9.47E-03 |
| TRIM67 | 10.9780 | 2.3441 | -2.2275 | 6.42E-18 | 5.12E-17 |
| BTBD17 | 31.0320 | 14.9407 | -1.0545 | 7.44E-25 | 1.58E-23 |
| TGIF1 | 1.4586 | 3.0382 | 1.0587 | 1.60E-20 | 1.80E-19 |
| MEOX2 | 0.1480 | 4.0000 | 4.7566 | 3.61E-08 | 8.87E-08 |
| MIR6071 | 5.3440 | 2.1719 | -1.2990 | 2.34E-22 | 3.46E-21 |
| MEGF10 | 2.4982 | 5.2613 | 1.0746 | 1.03E-14 | 5.44E-14 |
| MT1M | 5.4241 | 19.2254 | 1.8256 | 2.84E-19 | 2.70E-18 |
| IGLC2 | 0.9178 | 5.9752 | 2.7028 | 7.60E-07 | 1.64E-06 |
| AL390755.1 | 4.6246 | 11.6573 | 1.3338 | 1.18E-14 | 6.19E-14 |
| AC104072.1 | 2.3401 | 5.6661 | 1.2758 | 9.85E-07 | 2.10E-06 |
| COL23A1 | 1.0743 | 2.4761 | 1.2047 | 7.66E-06 | 1.48E-05 |
| IGKV3-11 | 0.3165 | 2.0507 | 2.6957 | 2.32E-08 | 5.83E-08 |
| SLIT2 | 0.7201 | 1.5565 | 1.1119 | 3.98E-02 | 4.85E-02 |
| TCEAL6 | 4.3733 | 8.9667 | 1.0359 | 9.28E-06 | 1.78E-05 |
| F3 | 13.5413 | 41.4629 | 1.6145 | 3.67E-28 | 1.31E-26 |
| SEMA3E | 0.5150 | 1.6124 | 1.6465 | 5.31E-11 | 1.80E-10 |
| CBR1 | 15.4861 | 36.3289 | 1.2301 | 1.15E-22 | 1.80E-21 |
| RDH10 | 2.3355 | 6.1436 | 1.3953 | 8.34E-13 | 3.51E-12 |
| EMILIN1 | 5.3534 | 11.1491 | 1.0584 | 3.87E-15 | 2.15E-14 |
| ALOX5AP | 7.4722 | 15.1484 | 1.0196 | 1.39E-18 | 1.20E-17 |
| NR2E1 | 2.1440 | 5.1526 | 1.2650 | 1.45E-07 | 3.34E-07 |
| DYNLT3 | 3.5987 | 7.7276 | 1.1025 | 3.48E-18 | 2.87E-17 |
| NWD1 | 1.2119 | 2.8091 | 1.2128 | 2.35E-09 | 6.59E-09 |
| KIF26A | 4.1785 | 1.9310 | -1.1137 | 1.97E-31 | 1.30E-29 |
| SFRP5 | 0.2702 | 2.0218 | 2.9033 | 1.16E-02 | 1.52E-02 |
| RAB34 | 2.0269 | 8.2504 | 2.0252 | 2.19E-33 | 2.15E-31 |
| HS6ST3 | 1.3001 | 2.7676 | 1.0900 | 1.05E-03 | 1.58E-03 |
| CCN1 | 14.4599 | 31.4111 | 1.1192 | 9.85E-08 | 2.32E-07 |
| FGFR3 | 14.0814 | 39.8748 | 1.5017 | 8.49E-21 | 9.99E-20 |
| GRIK1 | 1.0317 | 3.7980 | 1.8803 | 2.17E-10 | 6.87E-10 |
| TGFB2-AS1 | 1.8399 | 3.9765 | 1.1119 | 1.33E-21 | 1.76E-20 |
| KLRC4 | 2.7842 | 1.2244 | -1.1852 | 3.39E-34 | 4.04E-32 |
| RBPMS | 0.6983 | 1.4546 | 1.0587 | 7.23E-19 | 6.45E-18 |
| AC243562.2 | 2.7945 | 1.3588 | -1.0403 | 1.28E-26 | 3.63E-25 |
| GDNF-AS1 | 2.0911 | 0.5734 | -1.8666 | 1.01E-61 | 1.34E-57 |
| PCDH15 | 6.4118 | 3.0328 | -1.0800 | 1.73E-31 | 1.16E-29 |
| LAMB1 | 2.5291 | 6.0649 | 1.2619 | 5.39E-08 | 1.30E-07 |
| AC126407.1 | 3.1943 | 1.3910 | -1.1994 | 2.39E-33 | 2.31E-31 |
| CYP1B1 | 1.3010 | 2.8470 | 1.1298 | 7.90E-03 | 1.06E-02 |
| CLCF1 | 0.4497 | 1.8142 | 2.0123 | 4.56E-12 | 1.75E-11 |
| FZD6 | 1.0146 | 2.1772 | 1.1015 | 4.24E-09 | 1.16E-08 |
| CCDC8 | 0.5067 | 2.5341 | 2.3222 | 5.04E-20 | 5.23E-19 |
| SLC43A3 | 1.2253 | 3.0394 | 1.3107 | 6.79E-16 | 4.15E-15 |
| CHAC1 | 0.7533 | 1.8468 | 1.2937 | 1.98E-17 | 1.48E-16 |
| AC025211.1 | 2.2683 | 0.8352 | -1.4413 | 4.51E-40 | 2.49E-37 |
| SHD | 64.6747 | 27.8506 | -1.2155 | 2.60E-39 | 1.04E-36 |
| RSAD2 | 2.1863 | 4.5440 | 1.0555 | 6.73E-05 | 1.17E-04 |
| MTATP8P1 | 6.1427 | 2.6547 | -1.2103 | 6.23E-04 | 9.58E-04 |
| AC012213.4 | 3.6379 | 1.2575 | -1.5326 | 2.59E-26 | 6.96E-25 |
| COL8A2 | 1.3206 | 3.2845 | 1.3145 | 1.29E-13 | 5.99E-13 |
| KCNG1 | 0.7464 | 1.8767 | 1.3303 | 1.24E-08 | 3.21E-08 |
| AC092681.1 | 2.6940 | 1.2904 | -1.0619 | 7.16E-21 | 8.49E-20 |
| GNG12 | 6.7175 | 16.0897 | 1.2602 | 2.40E-26 | 6.53E-25 |
| EYA2 | 2.1563 | 5.1839 | 1.2655 | 5.98E-20 | 6.14E-19 |
| C7orf57 | 0.1930 | 2.0575 | 3.4139 | 9.62E-11 | 3.18E-10 |
| NOG | 12.8818 | 5.3134 | -1.2776 | 7.91E-34 | 8.50E-32 |
| C1R | 7.7087 | 25.8006 | 1.7428 | 2.76E-21 | 3.45E-20 |
| SLC8A3 | 5.0770 | 2.5067 | -1.0182 | 3.00E-24 | 5.77E-23 |
| SERPINF1 | 4.5591 | 14.9371 | 1.7121 | 3.76E-22 | 5.35E-21 |
| GALNT13 | 32.7379 | 15.9441 | -1.0379 | 1.12E-37 | 3.08E-35 |
| COLEC12 | 2.1156 | 4.9529 | 1.2272 | 2.35E-11 | 8.27E-11 |
| ANGPT1 | 0.5330 | 1.5585 | 1.5480 | 1.38E-15 | 8.05E-15 |
| ALDH1L1-AS2 | 1.1390 | 3.4664 | 1.6057 | 2.47E-19 | 2.38E-18 |
| DNAJA4 | 2.7059 | 5.4914 | 1.0211 | 1.09E-13 | 5.07E-13 |
| AL121894.2 | 2.5619 | 6.3156 | 1.3017 | 2.12E-14 | 1.08E-13 |
| OTOS | 0.2941 | 3.7557 | 3.6748 | 6.06E-29 | 2.51E-27 |
| XKR8 | 1.0014 | 3.2533 | 1.6999 | 8.70E-31 | 5.25E-29 |
| UPP1 | 2.0412 | 4.6118 | 1.1759 | 9.43E-27 | 2.73E-25 |
| SNHG18 | 0.5246 | 1.6064 | 1.6145 | 1.03E-09 | 3.01E-09 |
| CD44 | 15.4388 | 32.8058 | 1.0874 | 2.44E-14 | 1.23E-13 |
| AC007490.1 | 2.3077 | 0.9590 | -1.2669 | 2.65E-34 | 3.27E-32 |
| SOCS3 | 3.5529 | 14.0287 | 1.9813 | 1.12E-16 | 7.56E-16 |
| CTHRC1 | 0.6513 | 2.5625 | 1.9761 | 2.97E-08 | 7.38E-08 |
| AC093726.1 | 1.4933 | 3.4344 | 1.2016 | 3.45E-19 | 3.24E-18 |
| DDIT4L | 0.3136 | 3.0287 | 3.2717 | 5.11E-27 | 1.53E-25 |
| GSAP | 0.6974 | 1.5609 | 1.1623 | 1.36E-21 | 1.79E-20 |
| PCOLCE | 1.8655 | 4.7060 | 1.3349 | 3.81E-09 | 1.05E-08 |
| GJA1 | 116.7051 | 243.3264 | 1.0600 | 1.69E-18 | 1.44E-17 |
| CD163 | 4.3318 | 10.9567 | 1.3388 | 7.40E-04 | 1.13E-03 |
| MSN | 16.7450 | 44.2335 | 1.4014 | 2.65E-24 | 5.14E-23 |
| TLX1 | 2.0425 | 0.9020 | -1.1791 | 4.41E-18 | 3.60E-17 |
| IL1R1 | 0.9880 | 2.5163 | 1.3486 | 1.36E-10 | 4.39E-10 |
| EMP3 | 2.0597 | 18.4298 | 3.1615 | 3.76E-35 | 5.52E-33 |
| TGFB2 | 3.0931 | 8.5839 | 1.4726 | 3.88E-25 | 8.69E-24 |
| FOXJ1 | 2.8817 | 12.0892 | 2.0687 | 4.57E-06 | 9.03E-06 |
| RSPH4A | 0.4737 | 1.6060 | 1.7613 | 4.42E-12 | 1.70E-11 |
| AL118505.1 | 13.3399 | 5.3979 | -1.3053 | 7.61E-40 | 3.87E-37 |
| CTF1 | 1.0944 | 2.8832 | 1.3976 | 1.25E-32 | 1.06E-30 |
| NCR3LG1 | 1.5420 | 0.7551 | -1.0300 | 3.23E-15 | 1.81E-14 |
| MAN1C1 | 3.0736 | 7.7319 | 1.3309 | 4.26E-27 | 1.29E-25 |
| RRAD | 1.2930 | 2.7224 | 1.0741 | 2.47E-13 | 1.11E-12 |
| CST3 | 172.2062 | 363.7828 | 1.0789 | 9.77E-19 | 8.60E-18 |
| FSTL1 | 5.6715 | 15.2294 | 1.4251 | 3.58E-18 | 2.95E-17 |
| AL355974.2 | 1.8137 | 4.4512 | 1.2952 | 4.45E-09 | 1.21E-08 |
| CYP4F11 | 0.9300 | 1.9595 | 1.0751 | 2.49E-17 | 1.84E-16 |
| AL512625.2 | 4.3359 | 1.9949 | -1.1200 | 2.37E-31 | 1.50E-29 |
| GADD45A | 12.2763 | 25.4648 | 1.0526 | 3.73E-12 | 1.45E-11 |
| TYMP | 2.8491 | 6.1653 | 1.1137 | 4.22E-09 | 1.15E-08 |
| CACNG2 | 4.6777 | 1.8072 | -1.3720 | 1.41E-20 | 1.59E-19 |
| SLC13A3 | 0.9843 | 2.1041 | 1.0960 | 5.80E-15 | 3.16E-14 |
| LGI4 | 3.3699 | 11.0433 | 1.7124 | 1.65E-24 | 3.34E-23 |
| MIR9-3HG | 23.2503 | 11.0701 | -1.0706 | 1.49E-37 | 3.78E-35 |
| KLHL4 | 1.7764 | 3.7838 | 1.0909 | 5.65E-13 | 2.42E-12 |
| TCTEX1D1 | 0.1888 | 2.8542 | 3.9185 | 5.92E-25 | 1.28E-23 |
| MYO5C | 0.6404 | 1.3666 | 1.0937 | 9.97E-05 | 1.70E-04 |
| ACSS3 | 0.7277 | 2.1151 | 1.5393 | 1.24E-22 | 1.92E-21 |
| TMEM100 | 47.4449 | 22.4010 | -1.0827 | 6.88E-29 | 2.78E-27 |
| KLRC3 | 3.0078 | 1.2467 | -1.2706 | 1.01E-39 | 4.95E-37 |
| HLA-DRA | 143.6506 | 307.7091 | 1.0990 | 2.53E-13 | 1.13E-12 |
| MEGF11 | 10.9408 | 5.3050 | -1.0443 | 9.30E-33 | 8.15E-31 |
| S100A10 | 27.0000 | 61.1205 | 1.1787 | 7.18E-14 | 3.42E-13 |
| IGHG1 | 2.1785 | 6.3545 | 1.5444 | 1.37E-06 | 2.87E-06 |
| RPL39L | 0.9995 | 2.8050 | 1.4887 | 5.70E-25 | 1.24E-23 |
| IGKV3-20 | 0.4865 | 3.5838 | 2.8810 | 1.21E-04 | 2.03E-04 |
| TFCP2L1 | 0.3587 | 2.2502 | 2.6493 | 2.23E-14 | 1.13E-13 |
| COL4A2 | 7.4151 | 20.8566 | 1.4920 | 4.44E-05 | 7.87E-05 |
| TNC | 15.9207 | 38.5938 | 1.2775 | 2.06E-11 | 7.31E-11 |
| DIRAS3 | 1.7500 | 6.5831 | 1.9114 | 9.25E-26 | 2.31E-24 |
| TOMM40P4 | 3.7394 | 1.8295 | -1.0313 | 4.10E-32 | 3.19E-30 |
| LGI2 | 0.8399 | 2.2339 | 1.4112 | 2.05E-07 | 4.67E-07 |
| KCNE5 | 1.1638 | 6.1849 | 2.4100 | 1.07E-08 | 2.80E-08 |
| ANG | 0.5859 | 1.5263 | 1.3814 | 8.22E-29 | 3.29E-27 |
| ENTPD2 | 3.2956 | 7.3586 | 1.1589 | 1.11E-04 | 1.87E-04 |
| SERPINA5 | 0.1294 | 2.1570 | 4.0596 | 5.37E-23 | 8.85E-22 |
| LRRC61 | 0.5373 | 2.0476 | 1.9302 | 2.76E-19 | 2.63E-18 |
| RASGEF1A | 1.1835 | 2.4184 | 1.0310 | 7.70E-11 | 2.56E-10 |
| AC006960.1 | 1.7155 | 0.8018 | -1.0974 | 1.15E-20 | 1.33E-19 |
| CHST2 | 4.6302 | 9.4701 | 1.0323 | 3.31E-28 | 1.20E-26 |
| AC104051.2 | 20.1204 | 9.0120 | -1.1587 | 1.73E-08 | 4.43E-08 |
| NNMT | 0.5764 | 6.0448 | 3.3907 | 5.13E-27 | 1.53E-25 |
| BEST3 | 8.6893 | 4.0066 | -1.1169 | 3.05E-34 | 3.74E-32 |
| UNC79 | 5.8619 | 2.3270 | -1.3329 | 1.05E-28 | 4.12E-27 |
| ECRG4 | 1.0087 | 2.8533 | 1.5002 | 2.68E-16 | 1.72E-15 |
| TMEM176A | 3.9536 | 10.2372 | 1.3726 | 2.43E-22 | 3.57E-21 |
| CD74 | 184.2447 | 368.5345 | 1.0002 | 2.09E-15 | 1.20E-14 |
| AC015922.3 | 1.8027 | 4.0888 | 1.1815 | 1.57E-25 | 3.73E-24 |
| VWA1 | 6.1996 | 13.0876 | 1.0779 | 6.25E-21 | 7.48E-20 |
| LINC01445 | 0.4197 | 1.6358 | 1.9624 | 6.09E-03 | 8.29E-03 |
| SEZ6L | 78.4980 | 31.4166 | -1.3211 | 1.33E-28 | 5.14E-27 |
| MYO16 | 4.0605 | 1.9642 | -1.0477 | 1.77E-22 | 2.67E-21 |
| KCNIP2 | 26.8493 | 11.9251 | -1.1709 | 2.03E-12 | 8.16E-12 |
| FAM181A | 1.4781 | 5.6698 | 1.9395 | 1.42E-30 | 8.13E-29 |
| PI16 | 9.0005 | 18.2391 | 1.0190 | 5.40E-13 | 2.32E-12 |
| GABRA2 | 0.9478 | 2.0338 | 1.1015 | 2.11E-06 | 4.33E-06 |
| SPA17 | 1.1861 | 2.4823 | 1.0654 | 1.61E-23 | 2.81E-22 |
| IGFBP2 | 2.9329 | 24.6172 | 3.0693 | 1.31E-16 | 8.73E-16 |
| LRATD2 | 33.4930 | 16.3813 | -1.0318 | 1.91E-31 | 1.27E-29 |
| CA2 | 20.6268 | 42.8729 | 1.0555 | 7.08E-15 | 3.81E-14 |
| LINC01918 | 1.7447 | 0.7741 | -1.1725 | 2.05E-37 | 5.12E-35 |
| ARL4C | 8.4612 | 17.9252 | 1.0831 | 1.80E-11 | 6.42E-11 |
| LINC00092 | 0.6553 | 2.3764 | 1.8586 | 6.27E-19 | 5.65E-18 |
| NIBAN1 | 1.1942 | 2.6685 | 1.1600 | 8.79E-09 | 2.32E-08 |
| RGR | 6.4221 | 2.3600 | -1.4443 | 2.32E-31 | 1.48E-29 |
| SCN3A | 13.9423 | 6.1223 | -1.1873 | 2.23E-41 | 1.64E-38 |
| C3orf80 | 0.6530 | 1.3518 | 1.0498 | 2.57E-03 | 3.67E-03 |
| C5orf49 | 0.7229 | 3.4908 | 2.2717 | 1.69E-22 | 2.55E-21 |
| CA3 | 0.3959 | 4.2619 | 3.4284 | 4.60E-19 | 4.24E-18 |
| MRC2 | 6.0286 | 15.8522 | 1.3948 | 4.86E-19 | 4.46E-18 |
| PLBD1 | 0.7106 | 1.6226 | 1.1913 | 2.02E-16 | 1.31E-15 |
| NTS | 0.1074 | 3.1791 | 4.8872 | 1.14E-05 | 2.16E-05 |
| THEM5 | 0.7802 | 1.6918 | 1.1166 | 1.28E-07 | 2.98E-07 |
| HLA-DRB1 | 69.8268 | 155.1032 | 1.1514 | 8.73E-14 | 4.13E-13 |
| CD248 | 1.7567 | 4.5973 | 1.3879 | 2.75E-08 | 6.87E-08 |
| BST2 | 25.4759 | 54.6512 | 1.1011 | 1.20E-17 | 9.24E-17 |
| HLA-DRB6 | 5.0550 | 11.0577 | 1.1293 | 1.74E-08 | 4.43E-08 |
| GJB2 | 0.6952 | 2.2054 | 1.6656 | 4.28E-10 | 1.31E-09 |
| ABCC3 | 0.2195 | 2.2738 | 3.3726 | 1.68E-17 | 1.27E-16 |
| NBEAP2 | 7.9136 | 3.8213 | -1.0503 | 2.12E-37 | 5.19E-35 |
| LUM | 0.8740 | 2.9400 | 1.7501 | 1.68E-04 | 2.78E-04 |
| AL354919.2 | 0.4486 | 1.8150 | 2.0165 | 6.46E-08 | 1.54E-07 |
| ADM | 1.7558 | 4.1084 | 1.2265 | 2.37E-13 | 1.07E-12 |
| AC015922.2 | 2.1005 | 4.3983 | 1.0662 | 1.50E-17 | 1.14E-16 |
| FHDC1 | 4.6499 | 2.0529 | -1.1795 | 4.01E-25 | 8.96E-24 |
| GDAP1L1 | 23.9623 | 11.8074 | -1.0211 | 1.90E-27 | 6.20E-26 |
| SELENOM | 3.8391 | 8.0101 | 1.0610 | 3.06E-15 | 1.72E-14 |
| WIPF3 | 1.2297 | 3.5568 | 1.5322 | 1.01E-14 | 5.32E-14 |
| CHST8 | 1.1943 | 3.3502 | 1.4880 | 4.49E-06 | 8.87E-06 |
| HLA-DOA | 3.6667 | 8.1487 | 1.1521 | 4.37E-13 | 1.90E-12 |
| CD52 | 0.9646 | 1.9297 | 1.0004 | 2.66E-08 | 6.65E-08 |
| HVCN1 | 2.8639 | 5.8373 | 1.0273 | 2.75E-21 | 3.43E-20 |
| CDHR1 | 11.4688 | 4.4481 | -1.3665 | 3.11E-39 | 1.18E-36 |
| EVA1C | 1.4795 | 3.5901 | 1.2789 | 1.11E-29 | 5.31E-28 |
| SOCS2 | 1.1518 | 3.5843 | 1.6378 | 8.01E-16 | 4.85E-15 |
| KCNJ11 | 11.8062 | 5.5159 | -1.0979 | 2.46E-13 | 1.10E-12 |
| SOD3 | 1.6067 | 6.1533 | 1.9373 | 1.24E-27 | 4.17E-26 |
| PDGFD | 0.6468 | 1.7910 | 1.4693 | 1.13E-12 | 4.67E-12 |
| SPOCD1 | 1.2165 | 5.1972 | 2.0950 | 1.41E-09 | 4.07E-09 |
| SPRY1 | 2.0487 | 5.1139 | 1.3197 | 3.93E-08 | 9.61E-08 |
| TUBA1C | 2.1361 | 4.5580 | 1.0934 | 5.12E-10 | 1.55E-09 |
| FMOD | 1.1147 | 11.4343 | 3.3586 | 5.60E-11 | 1.89E-10 |
| OSMR | 1.6504 | 4.5154 | 1.4520 | 1.06E-16 | 7.16E-16 |
| CHI3L1 | 5.8011 | 208.2579 | 5.1659 | 2.64E-22 | 3.86E-21 |
| SMIM10 | 2.0196 | 4.2645 | 1.0783 | 1.06E-19 | 1.06E-18 |
| DYNLRB2 | 0.8259 | 1.8307 | 1.1483 | 8.03E-10 | 2.37E-09 |
| SCARNA9 | 1.6836 | 0.8405 | -1.0023 | 2.37E-22 | 3.50E-21 |
| NKD1 | 9.9363 | 4.4572 | -1.1566 | 3.17E-34 | 3.81E-32 |
| LINC02283 | 30.6381 | 12.0663 | -1.3443 | 2.91E-40 | 1.83E-37 |
| LRRN1 | 45.2996 | 22.5789 | -1.0045 | 4.51E-35 | 6.48E-33 |
| RGS16 | 2.4130 | 7.6231 | 1.6596 | 7.33E-23 | 1.18E-21 |
| LGALS1 | 50.2295 | 126.6349 | 1.3341 | 4.53E-36 | 8.32E-34 |
| AQP1 | 59.2800 | 244.2943 | 2.0430 | 1.14E-19 | 1.14E-18 |
| ANGPTL2 | 82.0035 | 37.5524 | -1.1268 | 6.45E-37 | 1.38E-34 |
| DSCAML1 | 13.5503 | 6.3635 | -1.0904 | 1.22E-42 | 1.47E-39 |
| SPX | 4.7023 | 12.0371 | 1.3561 | 1.07E-05 | 2.03E-05 |
| EPHA2 | 1.2993 | 2.6315 | 1.0182 | 7.14E-08 | 1.70E-07 |
| CAPS | 4.1758 | 8.9925 | 1.1067 | 1.43E-05 | 2.69E-05 |
| ARC | 11.8096 | 26.2043 | 1.1498 | 1.08E-08 | 2.82E-08 |
| RGMB | 10.8053 | 5.3986 | -1.0011 | 9.55E-36 | 1.62E-33 |
| H19 | 0.2977 | 5.7547 | 4.2726 | 2.38E-03 | 3.41E-03 |
| BATF3 | 0.8647 | 1.9063 | 1.1406 | 1.42E-16 | 9.39E-16 |
| KIAA1522 | 1.9184 | 4.0345 | 1.0725 | 9.23E-20 | 9.32E-19 |
| SOX8 | 250.9818 | 112.6574 | -1.1556 | 1.38E-41 | 1.14E-38 |
| COL5A1 | 0.5155 | 2.0330 | 1.9795 | 1.24E-07 | 2.89E-07 |
| HLA-DRB5 | 25.9057 | 55.6908 | 1.1042 | 1.67E-11 | 6.00E-11 |
| PDPN | 2.8282 | 19.2065 | 2.7637 | 4.96E-27 | 1.49E-25 |
| FOSL1 | 0.7252 | 2.2539 | 1.6359 | 1.88E-15 | 1.08E-14 |
| SLC17A8 | 3.6945 | 0.5815 | -2.6676 | 1.58E-10 | 5.07E-10 |
| IBSP | 0.3490 | 1.8304 | 2.3909 | 5.63E-07 | 1.23E-06 |
| IGLC3 | 0.6680 | 2.3629 | 1.8226 | 1.19E-04 | 2.00E-04 |
| PHETA2 | 0.7677 | 1.5712 | 1.0332 | 2.67E-20 | 2.88E-19 |
| FAM114A1 | 1.5687 | 3.6545 | 1.2201 | 2.29E-29 | 1.03E-27 |
| RORB | 1.8012 | 3.6105 | 1.0032 | 4.94E-04 | 7.69E-04 |
| CLDN10 | 1.7187 | 4.5972 | 1.4195 | 1.43E-09 | 4.12E-09 |
| OPLAH | 0.9395 | 2.5378 | 1.4336 | 3.80E-26 | 1.00E-24 |
| GSTM5 | 1.1145 | 3.3764 | 1.5991 | 6.71E-09 | 1.80E-08 |
| RGMB-AS1 | 1.4406 | 0.6496 | -1.1491 | 6.21E-39 | 2.16E-36 |
| CHGB | 41.4236 | 19.7623 | -1.0677 | 6.35E-18 | 5.08E-17 |
| SMIM18 | 7.6570 | 3.0928 | -1.3079 | 1.68E-33 | 1.69E-31 |
| LGI1 | 1.6247 | 3.4057 | 1.0678 | 1.17E-11 | 4.26E-11 |
| LINC00689 | 10.9454 | 5.3353 | -1.0367 | 9.24E-12 | 3.42E-11 |
| PRPS2 | 2.3616 | 4.9898 | 1.0792 | 2.25E-21 | 2.86E-20 |
| ZFP36 | 29.7384 | 61.0948 | 1.0387 | 6.78E-11 | 2.28E-10 |
| LZTS1 | 2.9585 | 6.3639 | 1.1051 | 1.41E-05 | 2.64E-05 |
| LINC02883 | 3.3859 | 1.5839 | -1.0961 | 2.90E-20 | 3.12E-19 |
| SLC6A11 | 6.1051 | 14.2203 | 1.2199 | 3.50E-04 | 5.54E-04 |
| NAPSB | 5.1319 | 11.5577 | 1.1713 | 2.23E-14 | 1.13E-13 |
| MX1 | 3.5663 | 9.3451 | 1.3898 | 6.61E-17 | 4.60E-16 |
| SDC4 | 11.0831 | 46.8304 | 2.0791 | 5.24E-33 | 4.75E-31 |
| PNPLA4 | 1.3044 | 3.3909 | 1.3782 | 2.91E-19 | 2.76E-18 |
| RIN1 | 0.8479 | 1.8824 | 1.1506 | 1.22E-15 | 7.18E-15 |
| ASIC4 | 32.8994 | 12.8308 | -1.3585 | 5.61E-30 | 2.90E-28 |
| HSPB1 | 47.9675 | 115.4217 | 1.2668 | 6.27E-25 | 1.35E-23 |
| DCTD | 6.8039 | 13.8559 | 1.0261 | 4.48E-35 | 6.48E-33 |
| RCOR2 | 29.7277 | 14.2145 | -1.0644 | 4.19E-37 | 9.73E-35 |
| EMILIN2 | 0.5378 | 2.0083 | 1.9009 | 3.31E-20 | 3.54E-19 |
| MT3 | 135.9499 | 294.9762 | 1.1175 | 7.33E-20 | 7.46E-19 |
| SERPINH1 | 4.8713 | 11.2021 | 1.2014 | 1.84E-12 | 7.45E-12 |
| STAC2 | 3.4842 | 7.0182 | 1.0103 | 2.82E-06 | 5.71E-06 |
| GFRA1 | 9.3330 | 3.6672 | -1.3477 | 8.84E-33 | 7.85E-31 |
| EMILIN3 | 0.9735 | 7.5972 | 2.9643 | 5.50E-17 | 3.87E-16 |
| ARSF | 0.6928 | 2.2586 | 1.7049 | 2.88E-12 | 1.14E-11 |
| FGFRL1 | 3.5618 | 10.2952 | 1.5313 | 7.96E-30 | 4.00E-28 |
| STEAP3 | 1.8073 | 7.2426 | 2.0027 | 1.02E-18 | 8.95E-18 |
| HSF2BP | 2.1535 | 1.0073 | -1.0962 | 6.11E-35 | 8.25E-33 |
| C1RL | 1.4443 | 3.9152 | 1.4387 | 4.19E-19 | 3.89E-18 |
| FABP7 | 21.6231 | 43.4303 | 1.0061 | 1.74E-05 | 3.22E-05 |
| SECTM1 | 0.8341 | 1.7182 | 1.0427 | 3.29E-06 | 6.61E-06 |
| CIITA | 0.6858 | 1.5983 | 1.2207 | 8.38E-11 | 2.78E-10 |
| FAM183A | 0.0954 | 2.9560 | 4.9528 | 1.25E-17 | 9.59E-17 |
| SPHKAP | 7.0123 | 2.5960 | -1.4336 | 4.92E-32 | 3.76E-30 |
| IGKC | 2.6508 | 7.0411 | 1.4094 | 1.38E-09 | 3.99E-09 |
| HMGA1P7 | 2.6625 | 0.7225 | -1.8817 | 6.44E-34 | 7.01E-32 |
| CHI3L2 | 4.7590 | 20.6615 | 2.1182 | 9.54E-16 | 5.70E-15 |
| FBLN5 | 2.4847 | 5.7466 | 1.2096 | 1.08E-15 | 6.40E-15 |
| SLC39A12 | 2.3883 | 5.8879 | 1.3018 | 9.40E-16 | 5.63E-15 |
| CPXM2 | 0.4850 | 1.7254 | 1.8310 | 4.09E-10 | 1.25E-09 |
| PLN | 3.7748 | 1.5777 | -1.2586 | 2.23E-02 | 2.81E-02 |
| PLP2 | 6.0356 | 16.9435 | 1.4892 | 5.16E-18 | 4.16E-17 |
| PLPPR1 | 35.9231 | 17.2880 | -1.0551 | 1.67E-34 | 2.09E-32 |
| IGFBP3 | 9.3195 | 20.9587 | 1.1692 | 3.23E-08 | 8.00E-08 |
| PIRT | 1.6182 | 4.4935 | 1.4734 | 7.89E-16 | 4.78E-15 |
| COL1A2 | 3.2580 | 11.4251 | 1.8101 | 2.71E-03 | 3.86E-03 |
| HSPA7 | 0.4544 | 2.2527 | 2.3096 | 2.27E-18 | 1.92E-17 |
| CFI | 2.7766 | 7.5296 | 1.4392 | 5.54E-10 | 1.67E-09 |
| PAMR1 | 3.9339 | 9.6886 | 1.3003 | 1.05E-16 | 7.09E-16 |
| TEX26 | 0.3690 | 1.6439 | 2.1554 | 1.87E-22 | 2.80E-21 |
| IL13RA2 | 0.8159 | 5.1526 | 2.6589 | 1.22E-11 | 4.43E-11 |
| CD109 | 0.6773 | 1.6993 | 1.3270 | 6.05E-17 | 4.24E-16 |
| FXYD1 | 1.0731 | 3.6183 | 1.7535 | 4.72E-17 | 3.36E-16 |
| CAVIN1 | 11.2925 | 28.0540 | 1.3128 | 3.19E-23 | 5.37E-22 |
| CCL5 | 1.0945 | 2.8086 | 1.3596 | 7.54E-11 | 2.51E-10 |
| PDLIM4 | 0.8546 | 6.0084 | 2.8137 | 2.98E-26 | 7.93E-25 |
| LINC02732 | 0.8247 | 1.7015 | 1.0449 | 5.57E-10 | 1.68E-09 |
| CSRP1 | 20.0551 | 42.2653 | 1.0755 | 7.31E-21 | 8.66E-20 |
| AL035446.1 | 0.7522 | 2.6136 | 1.7968 | 2.10E-07 | 4.77E-07 |
| ADGRE5 | 2.4731 | 5.2492 | 1.0858 | 9.98E-12 | 3.69E-11 |
| GJB6 | 2.6372 | 6.4799 | 1.2970 | 5.05E-03 | 6.94E-03 |
| DUSP5 | 1.8941 | 4.3414 | 1.1967 | 8.38E-12 | 3.12E-11 |
| AC009227.1 | 1.3894 | 0.6210 | -1.1618 | 2.94E-39 | 1.14E-36 |
| C9orf24 | 0.7664 | 4.3452 | 2.5032 | 3.26E-06 | 6.55E-06 |
| TEAD2 | 1.9100 | 3.8266 | 1.0024 | 1.36E-14 | 7.08E-14 |
| S100A6 | 119.1273 | 269.1086 | 1.1757 | 5.40E-20 | 5.58E-19 |
| AC062021.1 | 14.2342 | 3.7953 | -1.9071 | 9.38E-29 | 3.72E-27 |
| FAM110B | 30.0605 | 14.0963 | -1.0926 | 2.45E-42 | 2.70E-39 |
| ARSD | 1.4479 | 2.9266 | 1.0153 | 9.70E-13 | 4.06E-12 |
| CPQ | 3.0807 | 7.1404 | 1.2127 | 5.71E-29 | 2.38E-27 |
| MYH11 | 0.6940 | 1.5457 | 1.1552 | 2.30E-03 | 3.31E-03 |
| BHLHE40 | 10.1564 | 21.1745 | 1.0599 | 4.64E-25 | 1.03E-23 |
| IGLV1-47 | 0.6260 | 1.6276 | 1.3784 | 5.45E-05 | 9.57E-05 |
| GBP3 | 3.2745 | 6.7152 | 1.0362 | 7.12E-11 | 2.38E-10 |
| EFEMP2 | 2.8161 | 8.1004 | 1.5243 | 6.98E-25 | 1.49E-23 |
| DRAXIN | 1.3674 | 3.1586 | 1.2078 | 1.22E-02 | 1.59E-02 |
| SELENBP1 | 3.7532 | 8.6141 | 1.1986 | 6.36E-21 | 7.59E-20 |
| C7 | 0.9460 | 2.2396 | 1.2434 | 1.94E-03 | 2.82E-03 |
| IGFN1 | 3.0292 | 1.1769 | -1.3639 | 5.78E-16 | 3.56E-15 |
| G0S2 | 0.5399 | 3.5129 | 2.7018 | 1.50E-20 | 1.69E-19 |
| PNMT | 0.7030 | 2.5269 | 1.8457 | 1.14E-11 | 4.18E-11 |
| BRD9P2 | 1.3843 | 0.6876 | -1.0094 | 2.02E-18 | 1.71E-17 |
| RINL | 0.6812 | 1.4337 | 1.0736 | 1.05E-28 | 4.12E-27 |
| HSD11B1 | 1.4910 | 3.1664 | 1.0866 | 3.34E-03 | 4.70E-03 |
| P3H2 | 0.8885 | 2.2669 | 1.3513 | 1.13E-16 | 7.59E-16 |
| PLA2G2A | 0.1654 | 2.1976 | 3.7315 | 1.58E-16 | 1.04E-15 |
| LINC01116 | 0.6772 | 1.9369 | 1.5161 | 1.39E-02 | 1.81E-02 |
| CLIC1 | 14.5703 | 35.9647 | 1.3036 | 8.20E-21 | 9.65E-20 |
| RSPH1 | 1.8580 | 5.4905 | 1.5632 | 3.69E-20 | 3.91E-19 |
| IGHG3 | 0.3787 | 3.2543 | 3.1032 | 2.02E-05 | 3.73E-05 |
| AC023421.1 | 2.2168 | 0.8056 | -1.4603 | 7.19E-12 | 2.70E-11 |
| LINC02559 | 1.6227 | 0.5724 | -1.5033 | 7.18E-23 | 1.16E-21 |
| FAHD2B | 1.8139 | 3.7178 | 1.0354 | 6.51E-19 | 5.87E-18 |
| CELSR3 | 6.7650 | 2.9648 | -1.1902 | 6.88E-29 | 2.78E-27 |
| MXRA5 | 0.6823 | 1.4325 | 1.0702 | 2.36E-02 | 2.97E-02 |
| B3GNT5 | 0.5986 | 1.4023 | 1.2280 | 1.88E-17 | 1.41E-16 |
| PLA2G5 | 0.7733 | 5.0940 | 2.7196 | 1.06E-26 | 3.04E-25 |
| TENM3 | 0.6106 | 1.5337 | 1.3286 | 1.19E-06 | 2.52E-06 |
| C9orf64 | 1.6012 | 4.1680 | 1.3802 | 1.85E-24 | 3.70E-23 |
| MEIS3P1 | 1.1584 | 2.3658 | 1.0302 | 4.19E-14 | 2.05E-13 |
| SCRT1 | 10.7715 | 5.0787 | -1.0847 | 1.24E-20 | 1.43E-19 |
| CARD16 | 0.6567 | 1.8073 | 1.4605 | 7.68E-17 | 5.29E-16 |
| AL512329.2 | 0.4622 | 1.8551 | 2.0050 | 1.32E-19 | 1.31E-18 |
| CFAP52 | 0.6959 | 1.7426 | 1.3243 | 1.30E-02 | 1.70E-02 |
| KCNS1 | 1.0601 | 2.3924 | 1.1743 | 4.62E-03 | 6.39E-03 |
| TAGLN2 | 18.3320 | 56.9028 | 1.6341 | 9.93E-21 | 1.16E-19 |
| RAB36 | 0.9455 | 3.2167 | 1.7664 | 6.62E-22 | 9.20E-21 |
| SPP1 | 181.6960 | 397.8255 | 1.1306 | 1.21E-14 | 6.33E-14 |
| IGFBP7-AS1 | 0.8045 | 2.5466 | 1.6624 | 7.23E-05 | 1.25E-04 |
| SCRT2 | 2.2612 | 1.0690 | -1.0809 | 8.65E-17 | 5.92E-16 |
| FGFBP3 | 15.8850 | 7.6001 | -1.0636 | 2.03E-14 | 1.04E-13 |
| COL6A2 | 3.3066 | 13.4194 | 2.0209 | 5.05E-08 | 1.22E-07 |
| GLYCTK-AS1 | 1.5905 | 0.7749 | -1.0374 | 5.16E-30 | 2.68E-28 |
| FBLN1 | 2.5904 | 5.8696 | 1.1801 | 2.85E-15 | 1.60E-14 |
| ITGB4 | 9.8164 | 22.6735 | 1.2077 | 2.15E-21 | 2.74E-20 |
| ANXA1 | 5.8941 | 30.1895 | 2.3567 | 3.48E-18 | 2.87E-17 |
| AC012213.1 | 1.8570 | 0.5319 | -1.8037 | 2.61E-33 | 2.50E-31 |
| LAMA2 | 1.4311 | 3.0048 | 1.0701 | 5.02E-09 | 1.36E-08 |
| TNFAIP6 | 0.4934 | 2.5405 | 2.3643 | 2.59E-21 | 3.24E-20 |
| APOL4 | 1.3266 | 3.9996 | 1.5921 | 1.82E-11 | 6.50E-11 |
| NNAT | 12.5917 | 41.4258 | 1.7181 | 1.90E-07 | 4.34E-07 |
| ASIC4-AS1 | 14.5716 | 5.1200 | -1.5090 | 1.86E-36 | 3.70E-34 |
| LINC00957 | 1.2829 | 2.5760 | 1.0057 | 6.26E-13 | 2.67E-12 |
| RTP5 | 20.3380 | 7.1959 | -1.4989 | 5.22E-26 | 1.34E-24 |
| DCX | 12.4538 | 5.8117 | -1.0995 | 3.32E-36 | 6.19E-34 |
| HOPX | 5.7104 | 15.9408 | 1.4810 | 9.23E-25 | 1.94E-23 |
| ZCCHC12 | 2.1888 | 5.1792 | 1.2426 | 4.22E-06 | 8.37E-06 |
| CISTR | 1.5837 | 0.5783 | -1.4534 | 2.14E-15 | 1.22E-14 |
| ZDHHC22 | 54.2634 | 23.4736 | -1.2089 | 2.50E-36 | 4.78E-34 |
| SCGB1D2 | 0.4021 | 1.6725 | 2.0565 | 4.46E-16 | 2.78E-15 |
| AC009902.3 | 0.6378 | 1.3817 | 1.1152 | 8.28E-11 | 2.75E-10 |
| ABCC8 | 9.1053 | 3.7322 | -1.2867 | 1.18E-17 | 9.15E-17 |
| PYGL | 3.5905 | 7.4214 | 1.0475 | 3.19E-17 | 2.33E-16 |
| LTF | 0.5562 | 27.1757 | 5.6105 | 7.32E-15 | 3.93E-14 |
| MYH7 | 11.0877 | 3.7293 | -1.5720 | 3.05E-25 | 6.89E-24 |
| NDNF | 1.1089 | 2.4185 | 1.1250 | 1.32E-07 | 3.06E-07 |
| MT1A | 0.5334 | 1.9766 | 1.8898 | 1.61E-16 | 1.06E-15 |
| FLNC | 2.5926 | 12.0117 | 2.2120 | 8.24E-25 | 1.74E-23 |
| CBLN4 | 0.9990 | 2.4541 | 1.2966 | 5.06E-04 | 7.87E-04 |
| L1CAM | 18.4368 | 8.9887 | -1.0364 | 4.49E-10 | 1.37E-09 |
| PLSCR1 | 3.4315 | 6.9405 | 1.0162 | 4.11E-14 | 2.02E-13 |
| KIF19 | 0.8875 | 1.8258 | 1.0407 | 1.77E-08 | 4.50E-08 |
| CRB2 | 2.7495 | 7.7070 | 1.4870 | 7.46E-22 | 1.03E-20 |
| AL133465.1 | 5.8858 | 2.6590 | -1.1464 | 8.94E-17 | 6.10E-16 |
| AL358216.1 | 1.2894 | 2.8070 | 1.1224 | 3.02E-07 | 6.77E-07 |
| HSPA1B | 16.7366 | 35.8470 | 1.0988 | 2.43E-15 | 1.38E-14 |
| SERPINA1 | 2.8563 | 5.8382 | 1.0314 | 1.21E-15 | 7.12E-15 |
| CNGA3 | 2.1553 | 5.4583 | 1.3406 | 1.03E-03 | 1.54E-03 |
| MIR3151 | 1.7282 | 3.7447 | 1.1156 | 7.96E-08 | 1.89E-07 |
| GPC5 | 1.9023 | 3.8638 | 1.0223 | 1.82E-06 | 3.75E-06 |
| OSM | 0.7771 | 1.5554 | 1.0011 | 2.82E-05 | 5.13E-05 |
| LGR6 | 0.2254 | 4.0164 | 4.1553 | 3.30E-17 | 2.41E-16 |
| AL121820.2 | 0.8992 | 2.8909 | 1.6848 | 2.29E-21 | 2.91E-20 |
| THBS1 | 0.9701 | 3.4770 | 1.8416 | 1.64E-04 | 2.72E-04 |
| AMIGO2 | 0.6397 | 2.1437 | 1.7447 | 1.61E-24 | 3.28E-23 |
| GRIK2 | 12.3409 | 6.0558 | -1.0271 | 5.82E-37 | 1.26E-34 |
| RUBCNL | 0.7144 | 2.0152 | 1.4961 | 1.18E-21 | 1.58E-20 |
| RGS6 | 1.0571 | 2.4138 | 1.1912 | 2.78E-17 | 2.04E-16 |
| PDLIM1 | 1.9340 | 5.9154 | 1.6129 | 2.10E-09 | 5.94E-09 |
| TEAD3 | 0.8020 | 1.9006 | 1.2448 | 6.28E-17 | 4.38E-16 |
| SOD2 | 11.0302 | 31.0018 | 1.4909 | 8.90E-23 | 1.42E-21 |
| TCEA3 | 0.5183 | 2.1989 | 2.0849 | 1.78E-28 | 6.83E-27 |
| MAP3K7CL | 0.4939 | 1.5899 | 1.6867 | 1.56E-15 | 9.05E-15 |
| GNG3 | 19.6126 | 41.1112 | 1.0678 | 2.81E-04 | 4.50E-04 |
| GPNMB | 4.8565 | 10.6189 | 1.1287 | 3.52E-08 | 8.66E-08 |
| IGHA1 | 1.2451 | 14.4612 | 3.5378 | 1.92E-05 | 3.55E-05 |
| AC074286.1 | 1.9975 | 0.6598 | -1.5980 | 3.82E-46 | 9.45E-43 |
| SLC11A1 | 1.6302 | 4.1048 | 1.3323 | 1.46E-13 | 6.75E-13 |
| CDS1 | 1.5949 | 3.5700 | 1.1624 | 1.17E-08 | 3.04E-08 |
| WDR38 | 0.2638 | 2.1156 | 3.0035 | 4.66E-15 | 2.56E-14 |
| IFITM3 | 55.2919 | 121.4967 | 1.1358 | 5.28E-25 | 1.16E-23 |
| SYNC | 0.5721 | 1.5520 | 1.4397 | 9.49E-16 | 5.68E-15 |
| HLA-DPB1 | 22.9339 | 50.3832 | 1.1355 | 1.96E-13 | 8.88E-13 |
| CAPG | 10.8591 | 26.0220 | 1.2608 | 1.04E-22 | 1.63E-21 |
| KIAA0040 | 1.7084 | 5.1621 | 1.5953 | 2.51E-13 | 1.13E-12 |
| SLC14A1 | 8.7064 | 20.7926 | 1.2559 | 3.11E-06 | 6.27E-06 |
| MORN5 | 1.4238 | 3.3253 | 1.2237 | 3.97E-09 | 1.09E-08 |
| TNR | 90.2119 | 41.4532 | -1.1218 | 1.57E-35 | 2.48E-33 |
| PLEKHA4 | 5.3634 | 14.3427 | 1.4191 | 4.85E-16 | 3.01E-15 |
| SLC16A4 | 2.6966 | 5.4737 | 1.0214 | 2.69E-13 | 1.20E-12 |
| CYP27A1 | 8.7353 | 17.9198 | 1.0366 | 5.57E-22 | 7.81E-21 |
| SLN | 3.6519 | 10.4030 | 1.5103 | 1.49E-05 | 2.79E-05 |
| HDHD3 | 1.6540 | 4.1802 | 1.3376 | 2.26E-28 | 8.50E-27 |
| AMH | 8.3740 | 4.1310 | -1.0194 | 3.65E-20 | 3.87E-19 |
| S100A4 | 2.5611 | 7.8452 | 1.6151 | 5.74E-19 | 5.22E-18 |
| FREM2 | 0.6813 | 1.4261 | 1.0657 | 6.46E-03 | 8.76E-03 |
| TNFRSF12A | 3.1130 | 13.8076 | 2.1491 | 5.84E-22 | 8.14E-21 |
| CARTPT | 0.5017 | 2.6018 | 2.3745 | 1.98E-03 | 2.88E-03 |
| S100A11 | 32.7757 | 76.2599 | 1.2183 | 7.30E-18 | 5.77E-17 |
| HLA-DQB1 | 4.9136 | 10.5053 | 1.0963 | 2.08E-10 | 6.59E-10 |
| ETNK2 | 2.3710 | 4.9659 | 1.0666 | 3.73E-12 | 1.45E-11 |
| SAA1 | 0.0807 | 4.5610 | 5.8213 | 1.05E-11 | 3.86E-11 |
| HLA-DQA1 | 2.5950 | 6.4794 | 1.3201 | 4.91E-10 | 1.49E-09 |
| MARVELD1 | 1.8967 | 4.2790 | 1.1738 | 2.54E-19 | 2.44E-18 |
| HAPLN1 | 7.0344 | 2.6806 | -1.3919 | 4.72E-23 | 7.84E-22 |
