# Supplementary figures and images for: 5-Methylcytosine Related LncRNAs Reveal Immune Characteristics, Predict Prognosis and Oncology Treatment Outcome in Lower-Grade Gliomas
Source: Front Immunol. 2022 Mar 3;13:844778. doi: 10.3389/fimmu.2022.844778 (PMC8927645; doi:10.3389/fimmu.2022.844778)

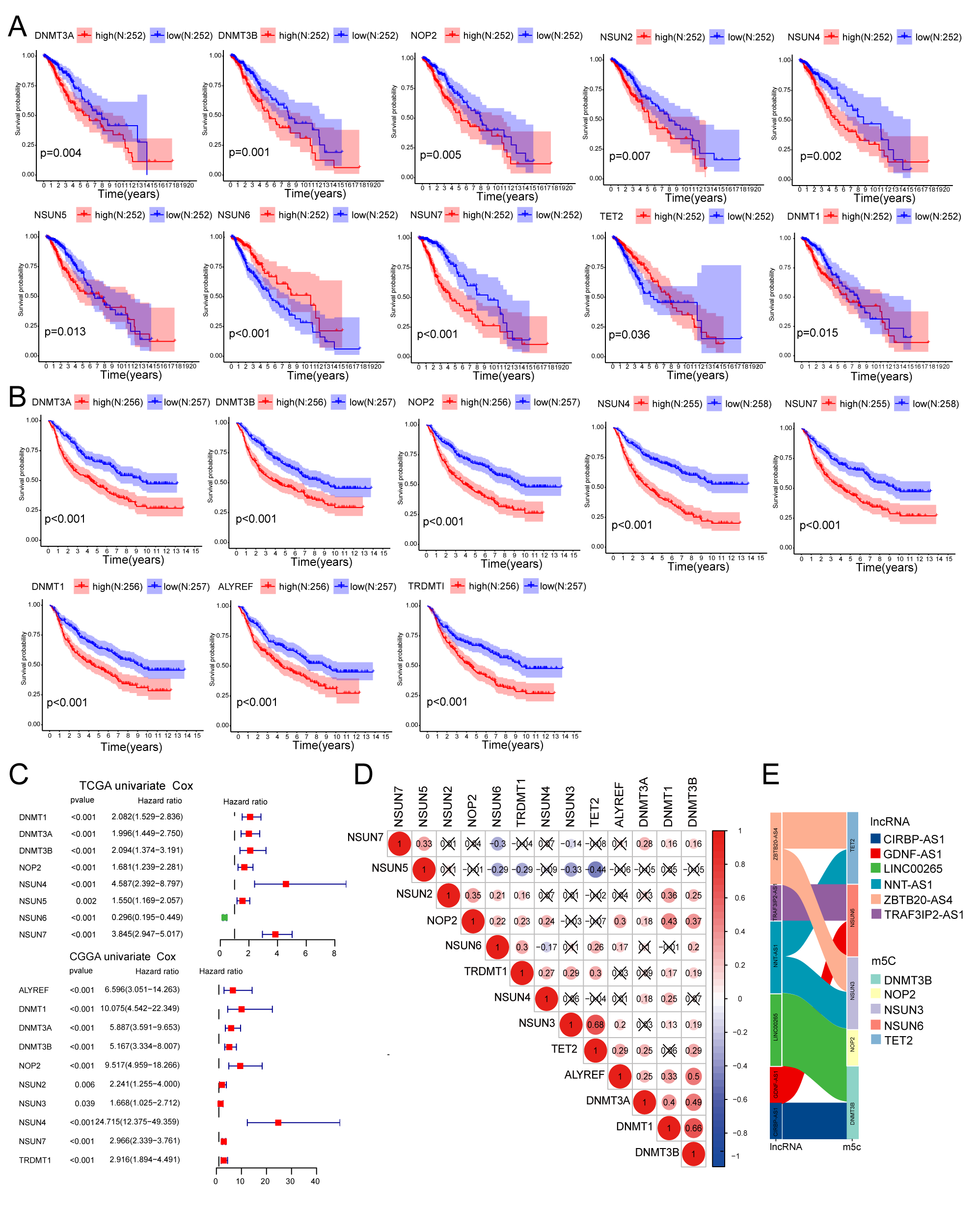

Supplement: Supplementary Figure 1 — K-M curves showing the survival differences between m5C regulators in the high- and low-expressed groups. The median expression was used as the cut-off. (A) the TCGA dataset, and (B) the CGGA dataset. (C) Univariate Cox regression results for 13 m5C regulators (p < 0.05 was exhibited). (D) Correlation between the 13 m5C methylation regulators based on TCGA dataset. (E) Correlation between LINC00265, CIRBP-AS1, GDNF-AS1, ZBTB20-AS4, NNT-AS1, TRAF3IP2-AS1, and corresponding m5C regulators based CGGA dataset. [file Image_1.tif]

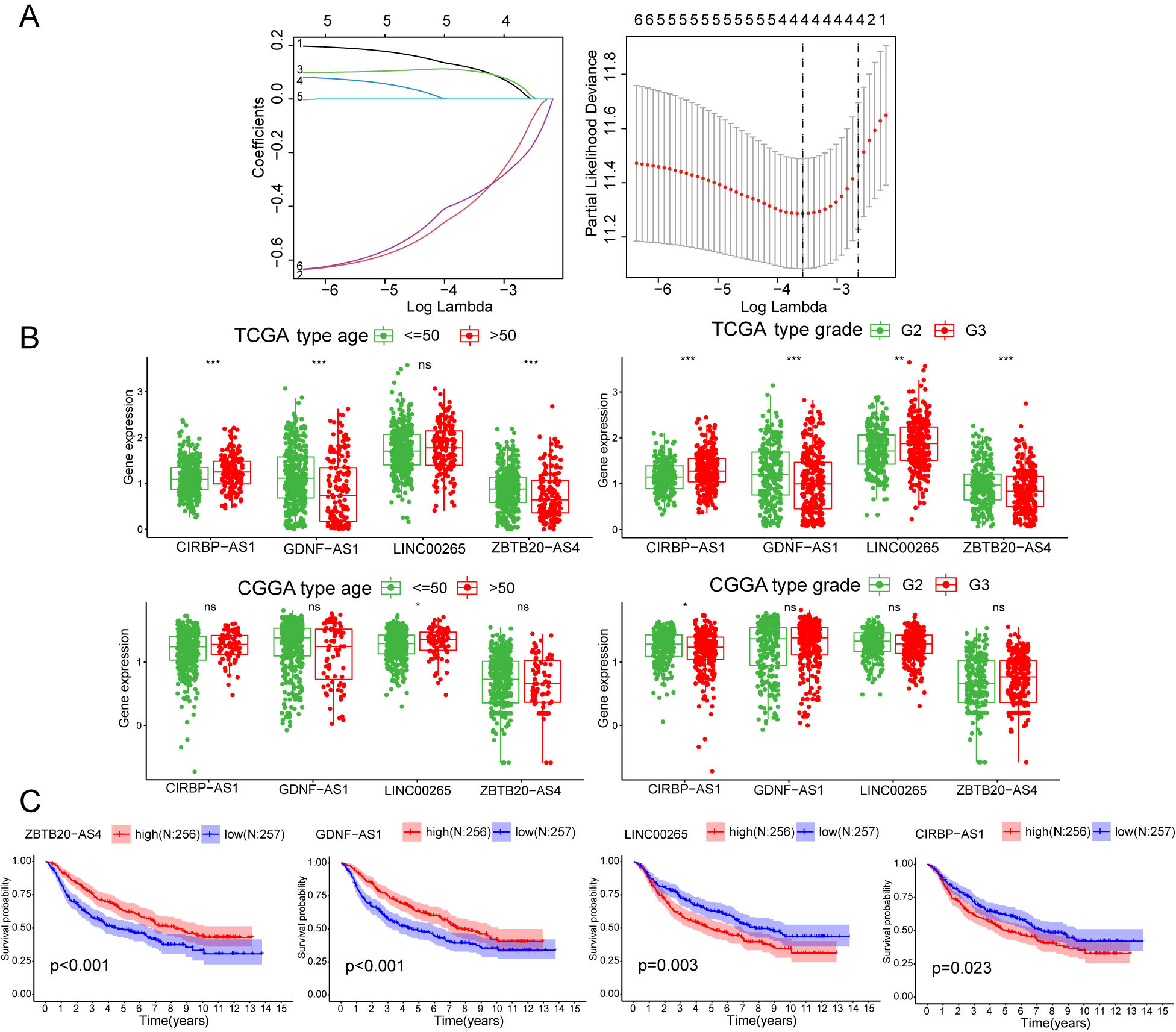

Supplement: Supplementary Figure 2 — (A) LASSO regression for 6 m5C-related lncRNAs based on TCGA dataset. (B) Differential expression of the 4 m5C-related lncRNAs in clinical subgroups (including WHO grade II or III, and age < =50 or >50 years). (C) K-M curves of the 4 m5C-related lncRNAs based on the CGGA dataset. (ns, non-significant, *p < 0.05, **p < 0.01, and ***p < 0.001). [file Image_2.tif]

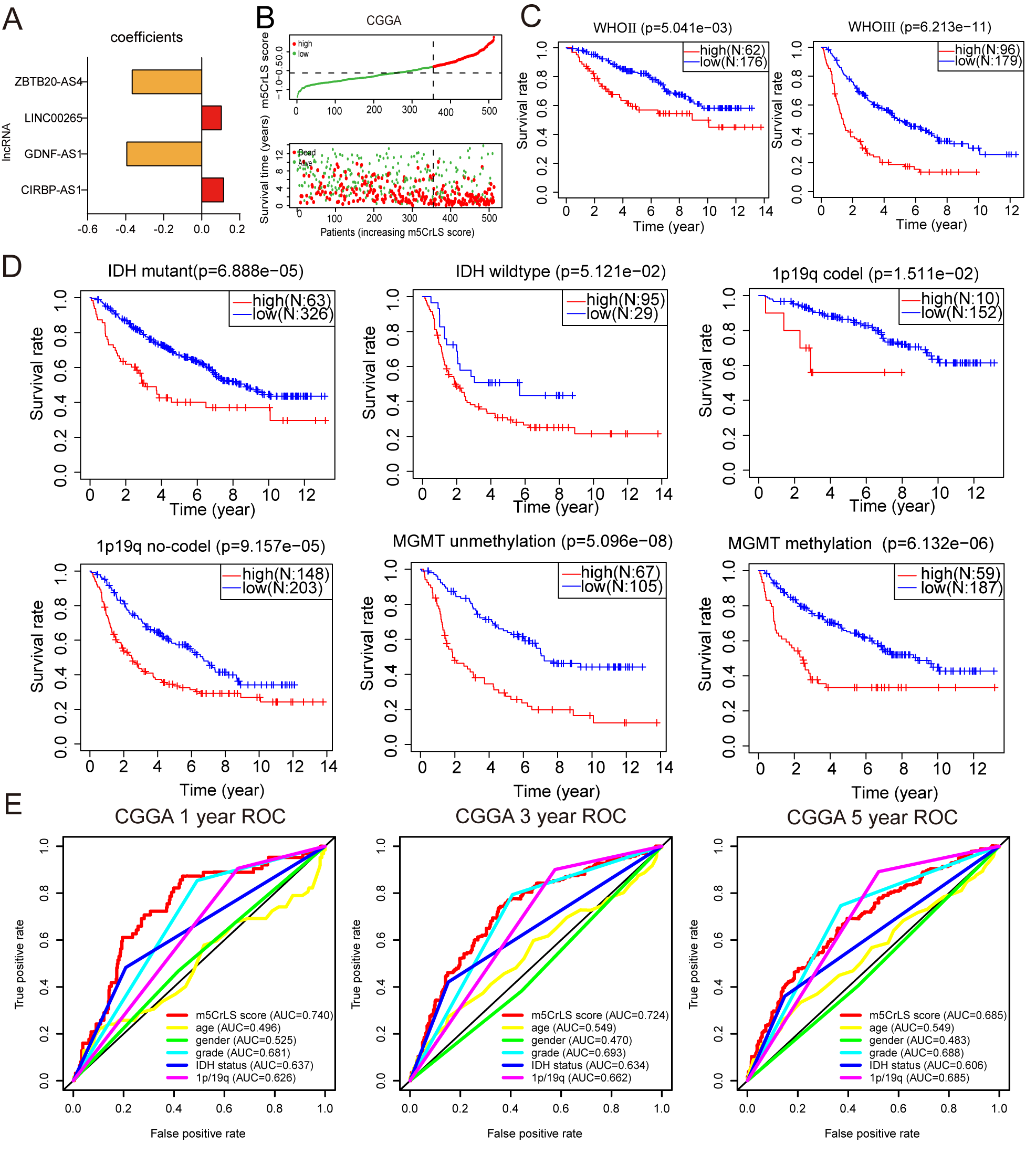

Supplement: Supplementary Figure 3 — (A) The LAASO regression coefficients of the 4 selected m5C-related lncRNAs. (B) The distribution plots of the m5CrLS score and survival status in the CGGA dataset. (C, D) K-M curves of the m5CrLS-based stratification in multiple CGGA clinical subgroups. (E) ROC curves were used to evaluate the prediction ability of m5CrLS score, age, gender, grade, IDH, and 1p/19q status (1-, 3-, and 5-year). [file Image_3.tif]

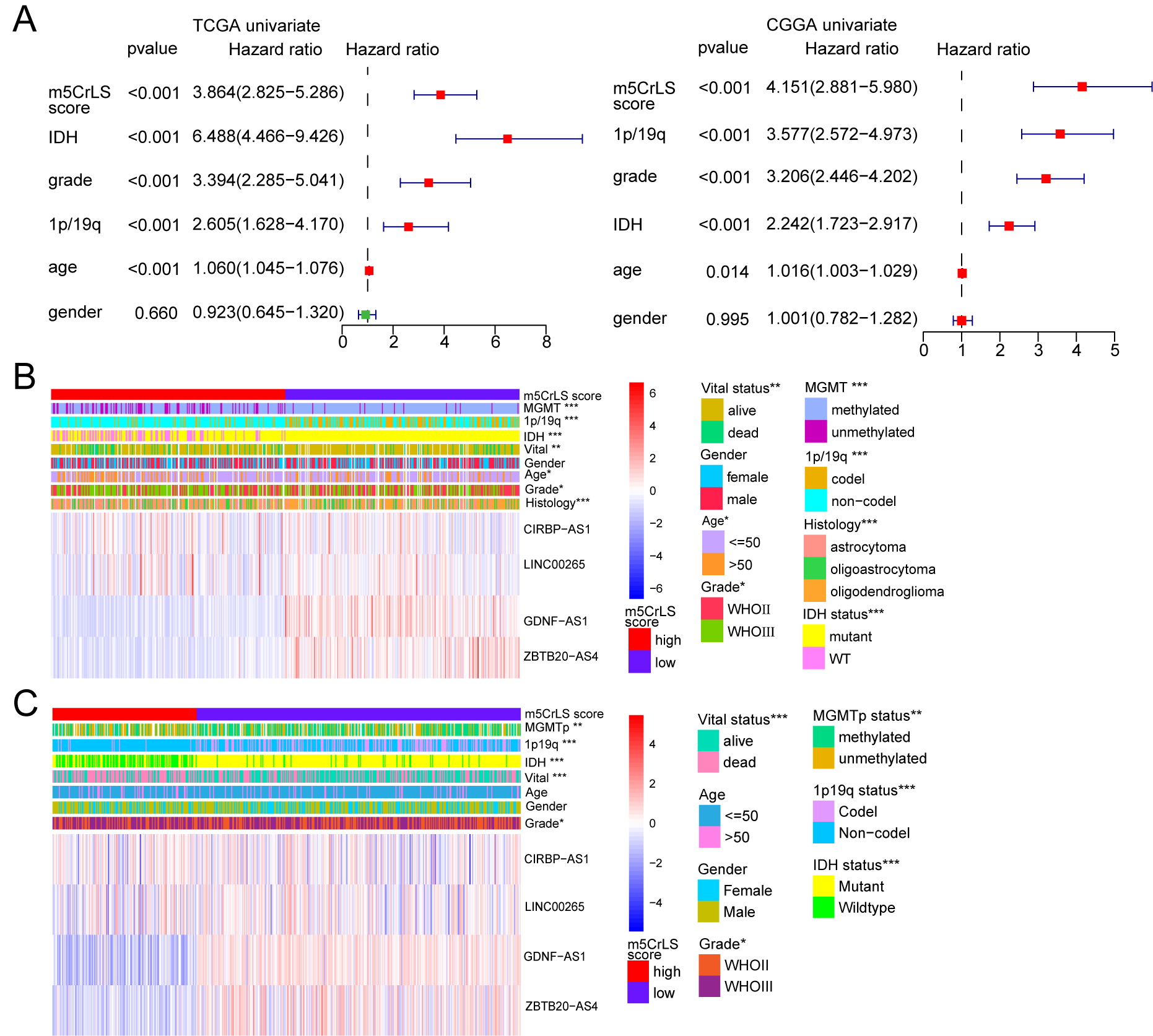

Supplement: Supplementary Figure 4 — (A) Univariate Cox regression analysis of the TCGA and CGGA datasets. Heatmap of the relationship between the m5CrLS score and clinicopathological features in the (B) TCGA and (C) CGGA datasets (*p < 0.05, **p < 0.01, and ***p < 0.001). [file Image_4.tif]

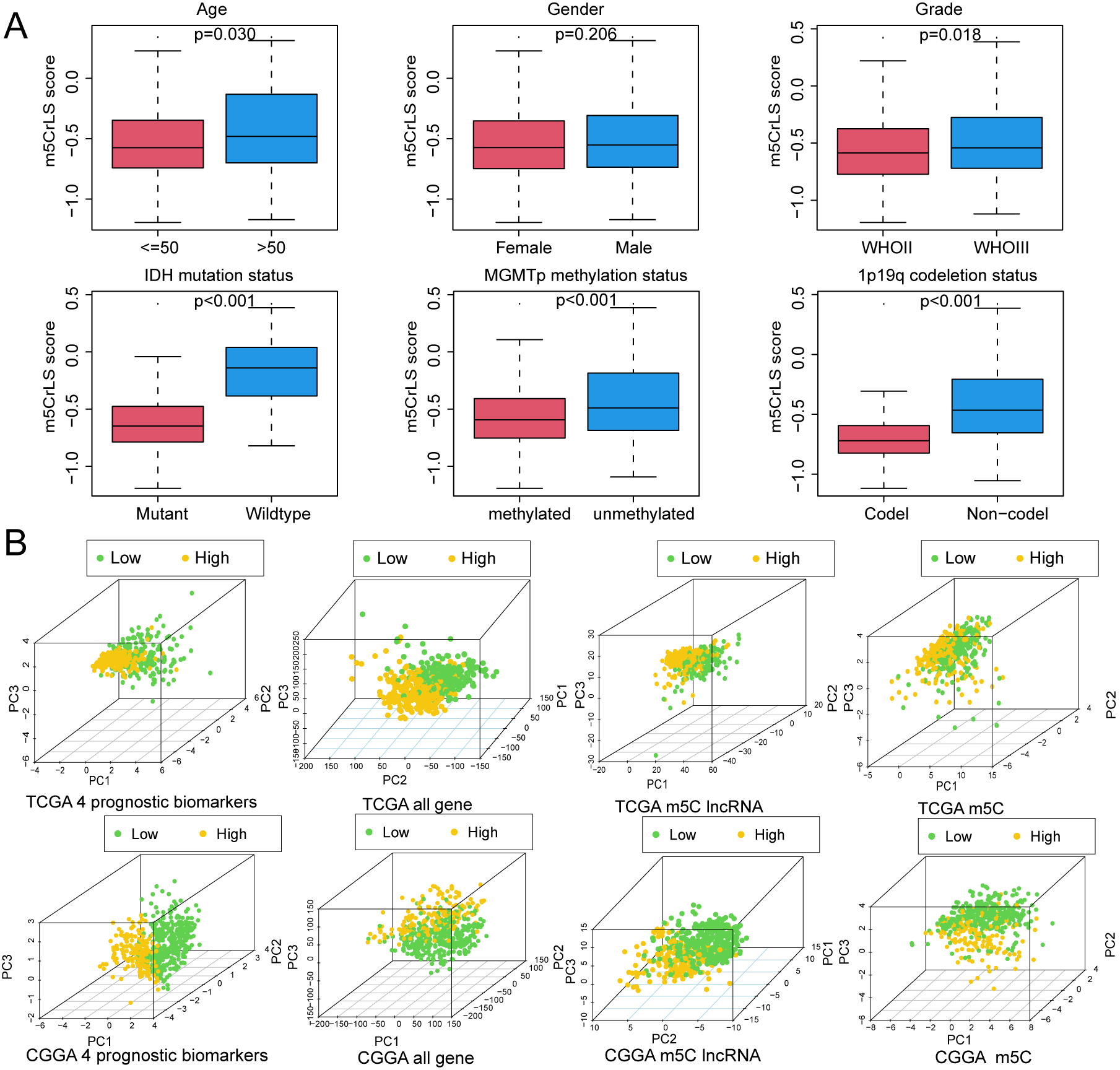

Supplement: Supplementary Figure 5 — (A) The m5CrLS scores of clinicopathological subgroups in the CGGA dataset. (B) The principal component analysis (PCA) of high and low m5CrLS score, based on the expression of the 4 prognostic biomarkers, all genes, m5C-related lncRNAs, and m5C regulators. [file Image_5.tif]

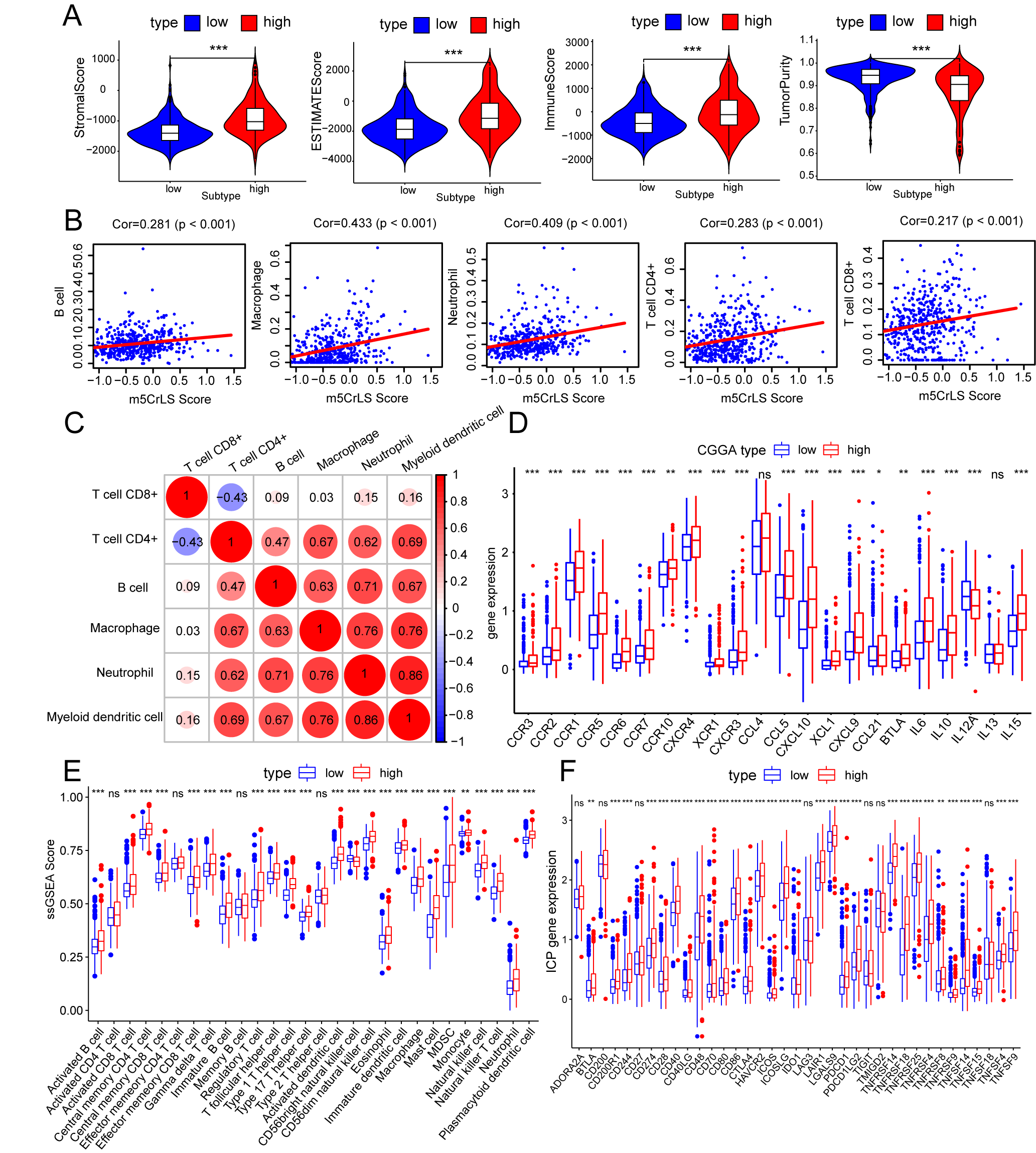

Supplement: Supplementary Figure 6 — (A) The stromal score, immune score, estimate score, tumor purity of CGGA dataset. (B) Correlation of 5 immune cells from TIMER2.0 with the m5CrLS score. (C) Spearman correlation between six tumor infiltrating cells. (D) Chemokines and cytokines associated with dendritic cells were differentially expressed between high and low m5CrLS score in CGGA dataset. (E) Differences in 29 immune cells between high and low m5CrLS score patients of CGGA dataset. (F) The expression level of ICPs in the CGGA dataset. (ns, non-significant, *p < 0.05, **p < 0.01, and ***p < 0.001). [file Image_6.tif]

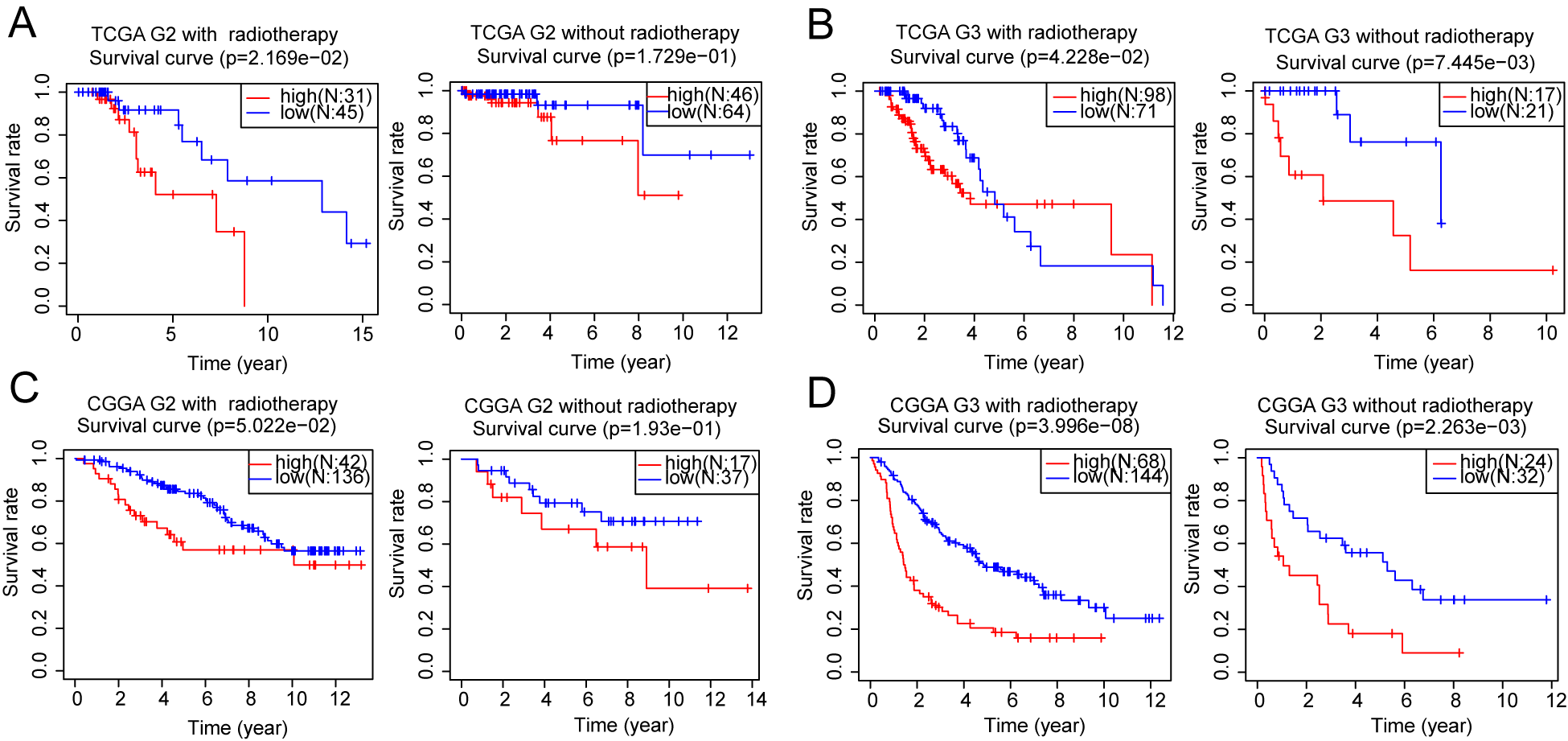

Supplement: Supplementary Figure 7 — K-M curves of (A) grade II and (B) grade III patients receiving radiotherapy or without radiotherapy based on TCGA dataset. K-M curves of (C) grade II and (D) grade III patients receiving radiotherapy or without radiotherapy based on CGGA dataset. [file Image_7.tif]
